# CS-DTA: a language model-driven framework for robust drug-target affinity prediction under strict cold-start scenarios

**DOI:** 10.3389/fchem.2026.1834317

**Published:** 2026-04-28

**Authors:** Zhaokun Jiang, Heying Dai, Yongxv Chen, Baixue Qiao, Shanwen Sun

**Affiliations:** College of Life Science, Northeast Forestry University, Harbin, China

**Keywords:** cold-start generalization, cross-modal interaction, drug-target affinity, large language models, out-of-distribution generalization

## Abstract

**Introduction:**

Accurate prediction of drug-target affinity is important for computational drug discovery, yet many deep learning models show limited robustness when applied to unseen compounds or previously uncharacterized proteins.

**Methods:**

We developed CS-DTA, a modular DTA prediction framework that integrates large language models (LLMs) for compound and protein representation learning with a cross-modal interaction module. By leveraging the strong transferability of LLM-based encoders, the model can capture rich semantic and structural patterns from both molecular sequences and protein sequences. The cross-modal attention mechanism further enhances prediction by explicitly modeling fine-grained interactions between compound and protein representations, enabling more effective fusion of complementary biochemical information.

**Results:**

CS-DTA achieved state-of-the-art predictive performance across warm-start and strict cold-start scenarios. Ablation analyses showed that the dual-encoder representation backbone was the primary source of predictive robustness. Interpretability analyses highlighted compact ligand substructures and localized protein regions with plausible relevance to binding. In downstream screening, CS-DTA prioritized known target-specific interactions, and selected predictions were supported by molecular docking. The non-kinase validation provided preliminary evidence of partial transferability beyond the kinase-focused benchmark setting.

**Discussion:**

The above results demonstrate that CS-DTA offers a robust and interpretable approach for DTA prediction. By integrating LLM-based encoding with a modular interaction architecture, the framework enhances generalization to previously unseen compounds and proteins while maintaining biologically meaningful interpretability. This combination makes CS-DTA a promising framework for virtual screening and early-stage drug discovery.

## 1 Introduction

The discovery of novel therapeutics is a multifaceted and resource-demanding process, fundamentally reliant on the identification of compounds that exhibit both high binding affinity and strict selectivity toward disease-associated targets (Sadybekov and Katritch, 2023; Sun et al., 2022). Central to this challenge is the accurate determination of drug-target affinity (DTA), which serves as a primary metric for evaluating the efficacy and safety profiles of potential drug candidates, since the landscape of known drug targets and empirical selectivity data consistently demonstrates that effective target engagement underpins successful clinical outcomes (Santos et al., 2017; Zeng et al., 2024). However, experimental determination of binding affinities remains time-consuming, labor-intensive, and costly, particularly during early-stage screening where millions of potential compounds must be evaluated (Cornaglia et al., 2017; Zeng et al., 2024). Moreover, the experimental coverage of chemical and proteomic space is inherently limited, restricting the ability to prioritize candidate compounds efficiently in large-scale discovery pipelines (Davis et al., 2011; Liu et al., 2025).

To address these limitations, computational methods for high-throughput screening have emerged as vital tools for accelerating drug discovery (Sadybekov and Katritch, 2023; Schneider, 2010). Traditional structure-based methods, such as molecular docking algorithms including AutoDock Vina (Trott and Olson, 2010) and Smina (Koes et al., 2013), aim to predict ligand binding poses and approximate binding energies based on three-dimensional structural information. While these methods have been widely adopted because they can accelerate hit identification relative to purely experimental screening, their performance remains significantly dependent on the availability and quality of three-dimensional (3D) target structures (Ferreira et al., 2015). Structural determination methods, such as X-ray crystallography, NMR spectroscopy, and cryo-electron microscopy (cryo-EM), often involve complex and time-consuming workflows in sample preparation, data acquisition, and structure refinement (Leelananda and Lindert, 2020). Moreover, although recent advances in protein structure prediction, particularly AlphaFold2 (Jumper et al., 2021), have substantially expanded structural coverage, docking-based methods remain limited by predictive structural uncertainty, difficulties in modeling conformational flexibility, and high computational cost in large-scale virtual screening (Díaz-Rovira et al., 2023; Pantsar and Poso, 2018; Scardino et al., 2023). These constraints highlight the need for complementary computational strategies that can estimate drug-target interactions (DTIs) more efficiently and generalize more reliably when high-quality bound structures are unavailable.

Recently, deep learning has expanded beyond screening-oriented prediction to applications such as biosynthetic design and biomedical association inference (Xie et al., 2024; Zhang Y. et al., 2025). Meanwhile, deep learning models have emerged as powerful alternatives for predicting DTA directly from molecular representations. Early architectures, exemplified by DeepDTA (Öztürk et al., 2018), transitioned the field from binary interaction classification to continuous affinity prediction by employing dual convolutional neural networks (CNNs) to extract latent features solely from 1D protein sequences and SMILES strings. However, because linear strings only partially reflect the intrinsic topological organization of small molecules, subsequent studies sought more structure-aware representations. GraphDTA, for example, advanced this lineage by representing drugs as molecular graphs. By utilizing graph neural networks (GNNs) to model atoms as nodes and bonds as edges, it effectively captured connectivity-driven features that are often obscured in string-based representations (Nguyen et al., 2020). To further incorporate mechanistic cues, later DTA research has increasingly pivoted toward explicit interaction modeling. MONN employed a multi-objective optimization strategy to concurrently learn global binding affinities and local, pairwise non-covalent interactions between atoms and residues (Li et al., 2020). This structure-free yet interaction-aware paradigm effectively reconciles the scalability of data-driven mapping with the interpretability of fundamental biochemical mechanisms. More recent DTA regression systems integrate these interaction-centric designs with large-scale pretrained representations. For instance, DTIAM unifies DTI, DTA, and mechanism prediction within a pretraining-finetuning framework, employing Transformer-based cross-modal attention over drug graphs and protein sequences (Lu et al., 2025). DTIAM demonstrates that interaction modeling benefits from enriched unimodal encoders, particularly in “cold-start” scenarios where either the drug or protein is previously unseen.

Despite these rapid advancements in deep learning-based DTA prediction, a formidable gap remains between benchmark performance and real-world utility. In real drug discovery, predictive models must frequently extrapolate to previously unseen compounds, previously unseen proteins, or both. Accordingly, robust performance under strict cold-start settings can be viewed as a practically important form of out-of-distribution (OOD) generalization (Ji et al., 2023). Earlier work had already emphasized that realistic evaluation of drug-target prediction should explicitly account for novel drugs and novel targets (Pahikkala et al., 2015). More recent studies likewise continue to frame cold-start prediction as a major unresolved challenge, and report marked performance degradation once evaluation is performed on unseen compounds or unseen proteins (Lu et al., 2025; Nguyen et al., 2022; Zhao Q. et al., 2025). Therefore, while modern architectures have introduced sophisticated cross-attention mechanisms (Chen et al., 2020; Qiao et al., 2024) or graph-based interactions to capture fine-grained compound-protein relationships (Huang et al., 2021; Li et al., 2020), their ability to deliver robust extrapolative performance under stringent cold-start conditions remains insufficiently resolved.

In this study, we present CS-DTA, a modular “Encode-Interact-Predict” framework specifically engineered for robust cold-start DTA prediction under a practically relevant OOD setting. By leveraging large language models (LLMs), namely, ESM2 (Lin et al., 2023) for proteins and ChemBERTa (Chithrananda et al., 2020) for small molecules, CS-DTA establishes an expressive foundation for small molecular and protein sequence representations. These unimodal features are subsequently aligned in a shared latent space and refined through a cross-modal interaction module to capture compound-protein complementarity. The resulting interaction-aware representations are then aggregated and passed to a feed-forward regression head to produce the final affinity prediction. To systematically evaluate its extrapolative capabilities, we benchmarked the proposed framework against representative state-of-the-art (SOTA) architectures (DeepDTA, GraphDTA, MONN, and DTIAM) across three rigorously enforced data splitting strategies: warm-start, protein-coldstart, and drug-coldstart. Through this comprehensive evaluation, we show that the combination of pretrained unimodal representations and a flexible interaction module yields strong predictive performance, with the clearest advantages observed under cold-start scenarios. In addition, we further assess the biological plausibility and practical relevance of the framework through perturbation-based interpretability analysis, molecular docking, and downstream virtual screening on an oncology kinase panel and a compact external validation on selected non-kinase targets.

The main contributions of this work are as follows:

- A modular framework for strict cold-start DTA prediction: We introduce CS-DTA, leveraging LLM-based encoders mapped to a shared latent space. By evaluating under separated OOD settings, CS-DTA demonstrates superior extrapolative capability, achieving SOTA predictive performance, particularly when generalizing to entirely unseen drug-like compounds and protein sequences.
- Interpretability-guided validation and downstream application: We validate the model’s physical plausibility through perturbation-based interpretability and molecular docking. Furthermore, application on an oncology kinase panel confirms that CS-DTA reliably recovers established target-specific pharmacological relationships, supporting its practical utility for high-throughput therapeutic prioritization.

## 2 Materials and methods

### 2.1 Datasets, entity-disjoint cold-start splits, and similarity-controlled evaluation

#### 2.1.1 Benchmark datasets and original entity-disjoint splits

To comprehensively evaluate the predictive performance and generalization capability of the CS-DTA framework, we utilized two widely recognized benchmark datasets: Davis et al. (2011) and KIBA (Tang et al., 2014). The Davis dataset is a kinase-focused affinity benchmark derived from a large-scale profiling study of kinase inhibitors, and the commonly used DTA version contains 30,056 interactions between 68 drugs and 442 targets, with binding strengths measured as dissociation constants ( $K_{d}$ ). In all Davis experiments, we used the standard ${pK}_{d}$ -transformed affinity values as the regression targets, where ${pK}_{d}$ was computed as $-\log_{10}\left(K_{d}\left[M\right]\right)$ .

The KIBA dataset is larger and more heterogeneous, because it integrates multiple kinase bioactivity measurements, including $K_{i}$ , $K_{d}$ and ${IC}_{50}$ , into a unified KIBA score. The filtered benchmark version widely adopted in deep learning studies contains 118,254 interactions involving 2,111 drugs and 229 targets. In all KIBA experiments, we directly used the benchmark-provided KIBA score as the training and evaluation target, rather than any single raw bioactivity measure.

To rigorously assess OOD generalization of our framework and prevent data leakage, we implemented an entity-disjoint cold-start splitting strategy. The datasets were partitioned into three distinct evaluation settings:

- Warm-start: The 8:1:1 splitting ratio is applied randomly to the drug-target interaction pairs. Both the drugs and proteins present in the test set share overlapping entities with the training set, representing a standard in-distribution evaluation.
- Protein-coldstart: The 8:1:1 partition is enforced at the level of unique protein entities. Proteins allocated to the test set are isolated and remain unseen during the training phase. This setting simulates the practical scenario of repurposing known drugs for novel or uncharacterized targets.
- Drug-coldstart: The 8:1:1 partition is enforced at the level of unique drug entities. Drugs allocated to the test set are excluded from the training set, mimicking the prospective virtual screening of novel chemotypes or newly synthesized compounds.

To ensure robust model optimization and prevent overfitting, the designated validation subset (10%) was strictly utilized for hyperparameter tuning, model selection, and early stopping. To eliminate sampling bias and ensure that the reported metrics reliably reflect the model’s predictive stability, this 8:1:1 partitioning process was independently repeated five times with different random seeds (a repeated hold-out validation strategy). All subsequent performance metrics are reported as the mean and standard deviation across these five independent runs.

#### 2.1.2 Audit of cross-split chemical similarity and sequence homology

To quantify the effective stringency of the released cold-start splits beyond simple entity disjointness, we performed an audit of residual cross-split chemical similarity on the drug axis and sequence homology on the protein axis. For the released drug-coldstart split, unique training-side compounds were defined as the union of compounds appearing in the training and validation subsets, and unique test-side compounds were extracted from the test subset. Canonical SMILES strings were retrieved from the ligand dictionary, parsed using RDKit, and converted to Bemis-Murcko scaffolds to evaluate scaffold-level overlap between training and test compounds. For each valid test compound, we further computed Morgan fingerprints (radius = 2, nBits = 2048) and identified its nearest neighbor in the training set using Tanimoto similarity. For each dataset and fold, we summarized the scaffold overlap rate together with the mean, median, and 95th percentile of the nearest-train similarity distribution; these statistics are reported in Supplementary Table S1. On the protein axis, unique training-side proteins were likewise defined as the union of training and validation proteins in the released protein-coldstart split, and unique test-side proteins were extracted from the held-out test subset. Protein sequences were written in FASTA format and compared using BLASTp, with the training proteins used to construct the search database. For each test protein, the nearest training homolog was selected as the best BLASTp hit, prioritizing alignments with query coverage of at least 70%; if no hit satisfied this coverage threshold, the overall best hit was retained. For each dataset and fold, we summarized the numbers of unique training and test proteins, the mean best-hit percent identity, and the proportion of test proteins whose nearest training hit exceeded 50% identity; these statistics are reported in Supplementary Table S2.

#### 2.1.3 Construction of similarity-controlled splits

To evaluate model robustness under more stringent out-of-distribution conditions, we constructed similarity-controlled cold-start splits on both the drug and protein axes. On the drug side, compounds were standardized at the SMILES level, and pairwise chemical similarity was computed using RDKit Morgan fingerprints (radius = 2, nBits = 2048) with Tanimoto similarity. Compounds were then grouped into similarity clusters by connecting pairs whose similarity exceeded a predefined threshold, and whole clusters were assigned to the training, validation, or test sets to avoid placing highly similar chemotypes across different splits. On the protein side, sequences were standardized and grouped into homology clusters according to pairwise global sequence identity, defined as the proportion of identical aligned residues over the full alignment length. Sequence pairs with identity above a predefined threshold were assigned to the same cluster, and whole clusters were then partitioned into the training, validation, and test sets. In both settings, cluster assignment was performed with a greedy procedure to approximately match the target pair-level split ratio. In this study, stricter drug similarity-controlled splits were constructed on Davis using thresholds of 0.30, 0.40, and 0.50, whereas stricter protein homology-controlled splits were constructed on KIBA using identity thresholds from 0.50, 0.60, and 0.70.

### 2.2 Model architecture of CS-DTA

The proposed CS-DTA framework comprises four key components: (1) Input representation and LLM encoding, (2) Unified latent projection, (3) Adaptable cross-modal interaction modeling, and (4) Aggregation and regression.

#### 2.2.1 Input representation and large language model encoding

Each DTA sample is represented as a pair $\left(S_{d},S_{p}\right)$ , where $S_{d}$ denotes the drug SMILES string and $S_{p}$ denotes the target amino-acid sequence. Both modalities are converted into token sequences: $x_{d}=\left[x_{d_{1}},\ldots ,x_{d_{L_{d}}}\right]$ for drugs and $x_{p}=\left[x_{p_{1}},\ldots ,x_{p_{L_{p}}}\right]$ for proteins, with $L_{d}$ and $L_{p}$ representing the respective token lengths.

During this tokenization phase, we enforce strict sequence length constraints to enable efficient mini-batch training. Specifically, protein sequences are padded or truncated to a uniform maximum length of $L_{p}^{\max}=1024$ , while drug sequences are strictly bounded to $L_{d}^{\max}=256$ . This preprocessing step directly outputs the standardized integer token sequences alongside their corresponding binary attention masks $m_{d}$ and $m_{p}$ . The modality-agnostic mask vector $m\in {\left\{0,1\right\}}^{L}$ is defined to distinguish valid tokens from padding elements:

$$m_{i}=\left\{\begin{array}{ll} 1, & \text{if token is valid}\\ 0, & \text{if token is padding} \end{array}\right.$$

Subsequently, the padded token sequences are directly fed into their respective LLM-based encoders. By processing these discrete tokens, ChemBERTa (Chithrananda et al., 2020) and ESM2 extract deep, context-aware representations, outputting the final-layer hidden states $H_{enc}\in R^{L\times h}$ , where h denotes the encoder-specific hidden size, and L is the bounded sequence length. The mask m is propagated alongside the hidden states to explicitly exclude padded positions in downstream computations, guaranteeing numerical consistency across variable-length samples.

#### 2.2.2 Unified latent projection

Since the drug and protein encoders may output different feature dimensions (i.e., $h_{d}\neq h_{p}$ ), learnable linear projections are applied to map both modalities into a shared latent space of dimension $d_{model}$ . This enables subsequent cross-modal attention to operate on a consistent feature scale. Given the pre-trained outputs $H_{d}\in R^{L_{d}\times h_{d}}$ and $H_{p}\in R^{L_{p}\times h_{p}}$ , the projected representations $Z_{d}$ and $Z_{p}$ are defined as:

$$Z_{d}=H_{d}W_{d}+b_{d},Z_{p}=H_{p}W_{p}+b_{p}$$

where $W_{d}\in R^{h_{d}\times d_{model}}$ and $W_{p}\in R^{h_{p}\times d_{model}}$ are learnable parameters, and $b_{d},b_{p}$ , are bias terms. We set $d_{model}=1024$ . The binary 1D masks $m_{d}$ and $m_{p}$ are preserved and propagated alongside **Z**_*d*_ and **Z**_*p*_ to exclude padded positions in downstream modeling and aggregation.

#### 2.2.3 Adaptable cross-modal interaction modeling

To capture complex ligand–target dependencies, we implement an adaptable cross-attention interaction module. To incorporate the 1D validity masks into the attention computation and strictly prevent information leakage from padded positions, additive mask matrices are constructed. When protein tokens serve as keys and values, the mask matrix $M_{p}\in R^{L_{d}\times L_{p}}$ is defined as:

$${\left(M_{p}\right)}_{i,j}=\left\{\begin{array}{ll} 0, & \text{if }{\left(m_{p}\right)}_{j}=1\\ -\infty , & \text{if }{\left(m_{p}\right)}_{j}=0 \end{array}\right.\;\left(i=1,\ldots ,L_{d};\;j=1,\ldots ,L_{p}\right)$$

Symmetrically, when drug tokens serve as keys and values, $M_{d}\in R^{L_{p}\times L_{d}}$ is constructed on $m_{d}$ . These additive masks are directly applied to the attention logits prior to the softmax activation.

We utilize Multi-Head Attention (MHA) to capture interaction features from complementary representation subspaces (Vaswani et al., 2017). The output of the *h*th attention head is computed via standard scaled dot-product attention:

$$head_{h}=S\text{oftmax}\left(\frac{\left(QW_{h}^{Q}\right){\left(KW_{h}^{K}\right)}^{T}}{\sqrt{d_{k}}}+M\right)\left(VW_{h}^{V}\right)$$

where $W_{h}^{Q}$ , ${\;W}_{h}^{K}$ , and $W_{h}^{V}$ are learnable projection matrices for the query ( Q ), key ( K ), and value ( V ) respectively, and *d*_*k*_ is the per-head dimensionality. The global multi-head output is obtained by concatenating all heads followed by a linear transformation $W^{O}$ :

$$\text{Mu}l\text{tiHead}\left(Q,K,V;M\right)=\text{Concat}\left({head}_{1},\ldots ,{head}_{H}\right)W^{O}$$

Within the full bidirectional variant of the CS-DTA framework, cross-attention is computed symmetrically. In the drug-to-protein ( d→p ) branch, drug representations act as queries to interrogate the protein context:

$$Z_{d\leftarrow p}=\text{MultiHead}\left(Z_{d},Z_{p},Z_{p};M_{p}\right)$$

Conversely, the protein-to-drug ( p→d ) branch updates protein features using the drug context:

$$Z_{p\leftarrow d}=\text{MultiHead}\left(Z_{p},Z_{d},Z_{d};M_{d}\right)$$

Crucially, this modular design allows the cross-attention pathways to be selectively configured (e.g., restricted to unidirectional or bypassed entirely). As demonstrated in our ablation studies, this adaptability serves as a structural regularization mechanism, preventing the model from learning spurious interaction shortcuts when evaluated on strictly out-of-distribution (OOD) novel compounds or targets.

#### 2.2.4 Aggregation and regression

To transform the variable-length, token-level representations into a unified, fixed-length embedding for affinity prediction, we apply a mask-aware mean pooling strategy. This step is crucial to ensure that the global pair representation is invariant to sequence length and strictly excludes artificial padding noise. Given a token-level feature matrix $Z={\left[z_{1},\ldots ,z_{L}\right]}^{T}\in R^{L\times d_{model}}$ and its corresponding binary validity mask $m\in {\left\{0,1\right\}}^{L}$ , the pooling operator is formally defined as:

$$\text{Pool}\left(Z,m\right)=\frac{\sum_{i=1}^{L}m_{i}z_{i}}{\sum_{i=1}^{L}m_{i}}$$

By normalizing over the sum of valid tokens, the resulting representations remain comparable and numerically stable. Depending on the specific architectural configuration, the relevant token matrices are pooled separately. For the bidirectional interaction variant, the interaction-aware matrices are pooled to obtain $v_{d}=\text{Pool}\left(Z_{d\leftarrow p},m_{d}\right)$ and $v_{p}=\text{Pool}\left(Z_{p\leftarrow d},m_{p}\right)$ . Notably, in the representation-driven baseline (where cross-attention is bypassed to enhance strict OOD generalization), the projected matrices $Z_{d}$ and ${\;Z}_{p}$ are pooled directly.

These modality-specific global vectors are subsequently concatenated to form a comprehensive fused representation:

$$r=\left[v_{d}∥v_{p}\right]{\in R}^{{2d}_{model}}$$

where “ ∥ ” denotes the concatenation operation. Finally, the fused embedding r is fed into a feed-forward multilayer perceptron (MLP) regressor to predict the continuous binding affinity score. The prediction is given by $\hat{y}=f_{\theta }\left(r\right)$ , where *θ* denotes the learnable parameters of the MLP.

The entire CS-DTA framework is optimized end-to-end by minimizing the Mean Squared Error (MSE) loss between the predicted affinities and the experimental ground-truth values. For a minibatch of N samples, the objective function is:

$$L_{MSE}=\frac{1}{N}\sum_{n=1}^{N}{\left(y_{n}-{\hat{y}}_{n}\right)}^{2}$$

where $y_{n}$ and ${\hat{y}}_{n}$ are the true and predicted affinities for the n *-th* sample, respectively. This objective effectively drives the network to capture predictive structural and physicochemical determinants governing drug-target binding.

### 2.3 Baseline models for comparison

To rigorously benchmark the predictive performance and out-of-distribution generalization of the CS-DTA framework, we compared it against four representative and SOTA deep learning architectures. These baselines were carefully selected to encompass diverse input representations and interaction modeling paradigms:

- DeepDTA: A classic sequence-based architecture that applies one-dimensional convolutional neural networks (1D-CNNs) to extract local representations from both drug SMILES strings and protein amino acid sequences (Öztürk et al., 2018).
- GraphDTA: A highly cited graph-based framework that converts drug SMILES into 2D molecular graphs processed by graph neural networks (GNNs), while retaining CNNs for protein sequence encoding (Nguyen et al., 2020).
- MONN: A multi-objective neural network that concurrently predicts non-covalent interactions and binding affinities by leveraging graph convolutional networks for ligands and CNNs for proteins (Li et al., 2020).
- DTIAM: A recent unified deep learning framework that employs large-scale self-supervised pre-training to learn informative substructures and contextual representations prior to affinity prediction (Lu et al., 2025).

To ensure a strictly fair and impartial comparison, all baseline models were implemented using their official open-source codebases and evaluated under the exact same experimental configurations as CS-DTA. Specifically, all models were trained and tested using the identical data splits (warm-start, protein-coldstart, and drug-coldstart) and evaluated via the same five repeated random splits, utilizing their recommended default hyperparameters.

### 2.4 Evaluation metrics

To quantitatively assess the predictive performance and ranking capability of the models across various evaluation splits, we employed four widely adopted evaluation metrics: Root Mean Square Error (RMSE), Pearson correlation coefficient ( R ), Spearman correlation coefficient ( ρ ), and Concordance Index (CI) (Gönen and Heller, 2005).

RMSE measures the average magnitude of the prediction errors, directly reflecting the absolute accuracy of the regression model. It is defined as:

$$\text{RMSE}=\sqrt{\frac{1}{N}\sum_{i=1}^{N}{\left(y_{i}‐{\hat{y}}_{i}\right)}^{2}}$$

where N is the total number of samples, $y_{i}$ represents the experimental ground-truth affinity, and ${\hat{y}}_{i}$ denotes the predicted affinity score. A lower RMSE value indicates superior predictive accuracy.

Pearson correlation coefficient ( R ) evaluates the linear correlation between the predicted and actual binding affinities:

$$R=\frac{\sum_{i=1}^{N}\left(y_{i}-\bar{y}\right)\left({\hat{y}}_{i}-\bar{\hat{y}}\right)}{\sqrt{\sum_{i=1}^{N}{\left(y_{i}-\bar{y}\right)}^{2}}\sqrt{\sum_{i=1}^{N}{\left({\hat{y}}_{i}-\bar{\hat{y}}\right)}^{2}}}$$

where $\bar{y}$ and $\bar{\hat{y}}$ are the mean values of the true and predicted affinities, respectively.

Spearman correlation coefficient ( ρ ) is a non-parametric measure of rank correlation, assessing how well the relationship between predictions and true values can be described using a monotonic function. It provides robust evaluation under distribution shifts (e.g., cold-start scenarios):

$$\rho =1-\frac{6\sum_{i=1}^{N}d_{i}^{2}}{N\left(N^{2}-1\right)}$$

where $d_{i}$ represents the difference between the rank of the predicted value and the rank of the true value for the i -th sample.

CI evaluates the model’s ability to correctly rank the binding affinities of paired drug-target interactions, which is particularly crucial for virtual screening campaigns. It computes the probability that the predicted values of two randomly selected pairs have the same relative order as their true values:

$$CI=\frac{1}{Z}\sum_{y_{i}>y_{j}}h\left({\hat{y}}_{i}-{\hat{y}}_{j}\right)$$

where Z is a normalization constant corresponding to the total number of valid pairs (i.e., $y_{i}>y_{j}$ ). The step function $h\left(x\right)$ is defined as:

$$h\left(x\right)=\left\{\begin{array}{ll} 1, & if\;x>0\\ 0.5, & if\;x=0\\ 0, & if\;x<0 \end{array}\right.$$

For Pearson ( R ), Spearman ( ρ ), and CI, higher values indicate better predictive performance and ranking consistency.

### 2.5 Interpretability and perturbation analysis

To characterize the local input regions that most strongly influenced representative model predictions, we performed a multi-view interpretability analysis combining sliding-window occlusion, SHapley Additive exPlanations (SHAP), and cross-attention visualization.

#### 2.5.1 Sliding-window occlusion on drug and protein axes

For a given drug-target pair, let $D=\left(d_{1},\ldots ,d_{L_{d}}\right)$ denote the tokenized drug sequence and $P=\left(p1,\ldots ,p_{L_{p}}\right)$ denote the tokenized protein sequence. Occlusion analysis was performed independently on the drug and protein axes using a sliding-window masking strategy. Specifically, for the drug axis, a contiguous window of length $w_{d}$ was moved along D with a fixed stride, and the tokens within the selected interval were replaced by a masking operator while the protein input P was kept unchanged. For the protein axis, the same procedure was applied to P using a protein-side window of length $w_{p}$ , while the drug input D was kept unchanged. In the implemented perturbation procedure, masked drug tokens were replaced with the tokenizer mask token (or the unknown token when no dedicated mask token was available), whereas masked protein residues were replaced with the character “X”. Accordingly, the drug-side and protein-side window lengths were set to $w_{d}$ = 15 and $w_{p}$ = 21, respectively, and the sliding stride was fixed to 1 in both scans.

Let $\hat{y}\left(D,P\right)$ denote the predicted affinity for the unperturbed input pair. For the i -th occluded window on the drug axis, the perturbed input is denoted by $D_{\text{occ}}^{\left(i\right)}$ , and the corresponding perturbation score is defined as

$$\Delta_{i}^{\left(d\right)}=\left|\hat{y}\left(D,P\right)-\hat{y}\left(D_{\text{occ}}^{\left(i\right)},P\right)\right|$$

Similarly, for the j -th occluded window on the protein axis, with perturbed input $P_{\text{occ}}^{\left(j\right)}$ , the perturbation score is

$$\Delta_{j}^{\left(p\right)}=\left|\hat{y}\left(D,P\right)-\hat{y}\left(D,P_{\text{occ}}^{\left(j\right)}\right)\right|$$

Thus, the impact of each local perturbation was quantified by the absolute change in predicted affinity relative to the original input. The resulting window-level scores were mapped back to the corresponding token or residue positions to generate drug-axis and protein-axis occlusion profiles, respectively. Regions associated with larger Δ values were interpreted as locally more influential to the model prediction.

#### 2.5.2 Segment-level SHAP analysis

To complement the occlusion-based sensitivity analysis, we applied SHAP on coarse-grained input segments along the drug and protein axes. For a given drug-target pair $\left(D,P\right)$ , the drug token sequence and protein token sequence were partitioned into contiguous segments, and each segment was treated as an interpretable feature. In the implemented analysis, the drug axis was partitioned into contiguous segments of four non-special drug tokens, whereas the protein axis was partitioned into contiguous segments of 20 residues. A binary coalition vector $z\in {\left\{0,1\right\}}^{M}$ was used to indicate whether each segment was retained or replaced by a masking operator, where M denotes the total number of segments on the analyzed axis. The model output under a given coalition was denoted by $f\left(z\right)$ , and the local additive explanation was written as

$$g\left(z\right)=\phi_{0}+\sum_{m=1}^{M}\phi_{m}z_{m}$$

where $\phi_{0}$ represents the baseline output and $\phi_{m}$ denotes the SHAP value of the m -th segment. Specifically, the baseline coalition z = 0 corresponded to an all-masked reference input on the analyzed axis. For the drug axis, all non-special drug tokens were replaced with the tokenizer mask token (or the unknown token when no dedicated mask token was available), while special and padding tokens were retained. For the protein axis, residues in excluded segments were replaced with the character “X”. Thus, $\phi_{0}=f\left(0\right)$ represented the model output under the corresponding fully masked baseline. The value $\phi_{m}$ quantifies the contribution of the corresponding local segment to the predicted affinity for the selected sample, with larger absolute values indicating stronger influence on the prediction. SHAP analysis was performed separately on the drug and protein axes, and the resulting segment-level attribution scores were projected back to the corresponding token or residue coordinates to generate the profiles shown in Figure 3. These attribution maps were then compared with the occlusion profiles to identify locally consistent high-contribution regions.

#### 2.5.3 Cross-attention extraction and score mapping

To visualize localized interaction patterns between the drug and protein inputs, cross-attention weights were extracted from the trained interaction module for representative predictions. For a given drug–target pair $\left(D,P\right)$ , let $L_{d}$ and $L_{p}$ denote the lengths of the drug and protein token sequences, respectively. For each attention head h , the drug-to-protein cross-attention matrix was denoted by

$$A^{\left(h\right)}\in R^{L_{d}\times L_{p}}$$

Where $A_{ij}^{\left(h\right)}$ represents the attention weight assigned from the i -th drug token to the j -th protein token. The final cross-attention map was obtained by averaging across heads,

$$\bar{A}=\frac{1}{H}\sum_{h=1}^{H}A^{\left(h\right)}$$

where H is the number of attention heads. Padding positions were excluded before visualization, and the resulting matrix $\bar{A}$ was rendered as a drug-token-by-protein-residue heatmap.

To facilitate biological and chemical interpretation, high-attention regions in $\bar{A}$ were mapped back to the original input coordinates. On the drug side, attention hotspots were aligned to the corresponding token intervals and then associated with the matched ligand substructures. On the protein side, the highlighted regions were mapped to the original residue indices of the input sequence. These mapped cross-attention hotspots were then compared with the occlusion- and SHAP-derived high-contribution regions to identify locally concordant interaction patterns.

### 2.6 Downstream application and virtual screening setup

To evaluate the practical utility of the CS-DTA framework in oncology drug repurposing, we established a systematic virtual screening pipeline. We curated a panel of 20 oncology-related key kinase targets. The target gene symbols were explicitly mapped to their reviewed, canonical UniProt accessions. To comply with the model’s maximum sequence length constraint ( $L_{p}\leq 1024$ ), sequences exceeding this limit were processed using a domain-aware extraction strategy. Specifically, canonical functional kinase domains were identified and extracted using UniProt annotations to ensure the structural retention of the primary binding pockets for small-molecule inhibitors, preventing the accidental cleavage of C-terminally located catalytic domains (The UniProt Consortium, 2025).

For the compound library, we retrieved FDA-approved small-molecule protein kinase inhibitors from the ChEMBL database (Zdrazil et al., 2024). Compounds were selected as maximum clinical phase 4 entries under the ATC code L01E, standardized into canonical SMILES, deduplicated based on their parent ChEMBL accession, and filtered by token length constraints, yielding a curated library of 197 approved inhibitors. The full list of compounds and corresponding identifiers is provided in Supplementary Table S7.

To effectively highlight target-specific prioritization and mitigate the visual interference of broadly active agents, we applied a visualization-oriented heuristic refinement procedure to the raw predictions. First, rapalog-class inhibitors (e.g., everolimus, sirolimus, and temsirolimus) were exclusively retained for the MTOR target and removed from the ranking lists of other kinases. Second, highly promiscuous compounds defined empirically as those appearing in the top 20 rankings across 10 or more different targets were excluded from the representative clean top-10 visualizations.

To establish an external evidence chain for our predicted positive controls (i.e., rediscovery validation), we systematically queried historical bioactivity records in the ChEMBL database using the corresponding target ChEMBL id. For highly ranked drug-target pairs, we extracted standard assay metrics (e.g., $IC_{50}$ , $K_{d}$ ) and reported the total number of activity records (number of activity records) alongside the maximum standardized pChEMBL value (max pChEMBL) as empirical validation of the model’s predictive reliability.

### 2.7 Molecular docking and structural visualization

To assess the physical and geometric plausibility of the model’s top-ranked interactions, focused molecular docking was performed using AutoDock Vina (Trott and Olson, 2010). The high-resolution co-crystal structure of the EGFR kinase domain (PDB ID: 4G5P) was selected as the receptor template (Burley et al., 2025). Receptor preparation was conducted using PyMOL to completely remove crystallographic water molecules and the original co-crystallized ligands. Subsequently, polar hydrogen atoms were added to the receptor structure using AutoDockTools. The 3D conformer of the selected ligand, afatinib, was obtained from the PubChem database in SDF format (Kim et al., 2025). To specifically sample the catalytic ATP-binding pocket, the docking grid box was constructed with its center coordinates strictly aligned to the geometric center of the original co-crystallized ligand. Finally, the optimal binding poses, spatial orientations, and drug-target interactions were visually analyzed and rendered using PyMOL.

### 2.8 Construction of an external non-kinase validation panel

To further examine whether CS-DTA retains ligand-ranking signals beyond kinase-centered benchmarks, we constructed a compact external panel centered on four oncology-relevant human non-kinase targets. Drug-target relationship data were collected from the IUPHAR/BPS Guide to PHARMACOLOGY (GtoPdb) (Harding et al., 2025), and protein sequences were obtained from The UniProt Consortium (2025). The selected targets included poly (ADP-ribose) polymerase 1 (PARP1), estrogen receptor alpha (ESR1), aromatase (CYP19A1), and androgen receptor (AR). These targets were chosen because they are supported by curated ligand annotations and span distinct non-kinase target classes relevant to oncology, including a DNA-repair-associated enzyme (PARP1), a steroidogenic enzyme (CYP19A1), and nuclear hormone receptors (ESR1 and AR). The full list of targets is provided in Supplementary Table S9.

Starting from the corresponding human target-ligand annotations in GtoPdb, we extracted ligand records associated with the four selected targets. Small-molecule structures were represented using canonical SMILES strings, and protein inputs were represented using the corresponding UniProt canonical sequences. The compound panel comprised 42 ligands and is provided in Supplementary Table S10. This non-kinase panel was subsequently used for pairwise scoring and rank-based external evaluation.

### 2.9 Implementation details

The CS-DTA framework was implemented using the PyTorch deep learning library (Paszke et al., 2019). The language model-based encoders ChemBERTa and ESM2 (35M parameter variant) models were used. During the preprocessing stage, the maximum sequence lengths were strictly truncated to 256 tokens for drugs and 1,024 tokens for proteins.

For model optimization, we employed the AdamW optimizer with a decoupled weight decay of $1\times 10^{-4}$ (Loshchilov and Hutter, 2017). To preserve the generalized representations captured by the LLMs while enabling effective task-specific adaptation, a differential learning rate strategy was implemented. Specifically, the LLM-based encoders were fine-tuned with a lower learning rate of $1\times 10^{-5}$ , whereas the randomly initialized cross-attention and regression modules were trained with a higher learning rate of $2\times 10^{-4}$ .

The models were trained for a maximum of 100 epochs using a mini-batch size of 32. To dynamically adapt the learning process, a ReduceLROnPlateau scheduler was adopted, which decayed the learning rate by a factor of 0.5 if the validation RMSE did not improve for 3 consecutive epochs. Furthermore, to prevent overfitting, an early stopping mechanism was enforced with a patience of 10 epochs based on validation performance. All training procedures were accelerated using PyTorch Automatic Mixed Precision (AMP) on NVIDIA GPUs to optimize computational efficiency and memory utilization.

## 3 Results

### 3.1 Architecture of the CS-DTA framework

**FIGURE 1:**
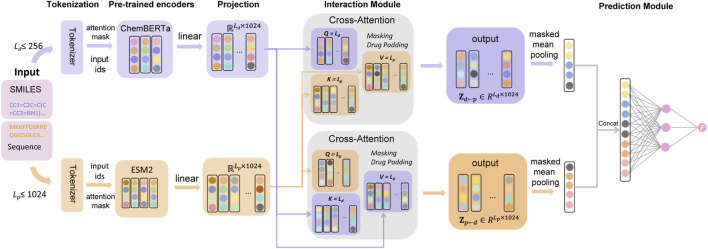
Overall architecture of the CS-DTA framework. Drug SMILES and protein sequences are tokenized and encoded by ChemBERTa and ESM2, respectively, then projected into a shared latent space. Cross-modal information is modeled by the interaction module, illustrated here using bidirectional cross-attention. The resulting drug and protein representations are pooled, concatenated, and passed to a feed-forward prediction module to estimate binding affinity.

As illustrated in Figure 1, we propose CS-DTA, a modular encode–interact–predict framework designed to address the central challenge of DTA prediction under strict cold-start settings. The core design principle is to decouple generalizable unimodal representation learning from task-specific cross-modal interaction modeling, resulting in two main components: a dual-domain LLM encoding module and a bidirectional cross-attention interaction module. Specifically, the framework integrates two SOTA LLMs—ChemBERTa (Chithrananda et al., 2020) for small molecules and ESM2 (Lin et al., 2023) for proteins. Large-scale pretraining enables these models to capture biochemical regularities and structural priors that are difficult to infer from benchmark-scale DTA datasets alone (Zhang H. et al., 2025). Such transferable priors provide a robust representational foundation, allowing the model to maintain strong extrapolation capability when encountering compounds or targets that are absent from the training set.

Building upon this aligned representation space, CS-DTA introduces a bidirectional cross-attention mechanism to model compound–protein interactions. Unlike simple feature concatenation, this module is designed to reflect the physicochemical complementarity between molecules and proteins. Through context-dependent feature refinement, drug representations are updated in the context of protein features, while protein representations are simultaneously conditioned on drug features. This design enables the model to capture bidirectional signals that are directly relevant to binding affinity, thereby learning binding-aware representations that more faithfully reflect the underlying mechanisms governing molecular interactions.

Finally, the encode-interact-predict decomposition was retained deliberately to make the contribution of interaction modeling directly testable. By separating pretrained representation extraction from cross-modal interaction refinement and affinity regression, CS-DTA provides a controlled framework in which the role of the interaction module can be examined explicitly rather than being entangled with the unimodal encoders. This modularity is important for interpreting the subsequent results, because it allows performance differences across warm-start and strict cold-start settings to be attributed more clearly to interaction modeling itself. Taken together, this architecture was designed not only to support accurate affinity prediction, but also to provide a transparent structural basis for evaluating how interaction-aware modeling influences generalization under distribution shift.

### 3.2 CS-DTA achieves state-of-the-art predictive robustness across both warm-start and cold-start scenarios

To comprehensively evaluate the extrapolative capabilities of CS-DTA, we benchmarked its performance against four representative SOTA models (DTIAM, GraphDTA, MONN, and DeepDTA) on both the Davis et al. (2011) and KIBA (Tang et al., 2014) datasets across three rigorous data splitting strategies: warm-start, protein-coldstart, drug-coldstart. In the warm-start setting, both test drugs and test proteins may also appear in the training set, but their paired interactions are unseen. In the protein-coldstart setting, all proteins in the test set are excluded from the training set. In the drug-coldstart setting, all drugs in the test set are excluded from the training set. Therefore, the latter two settings specifically evaluate model generalization to previously unseen targets or previously unseen compounds.

**FIGURE 2:**
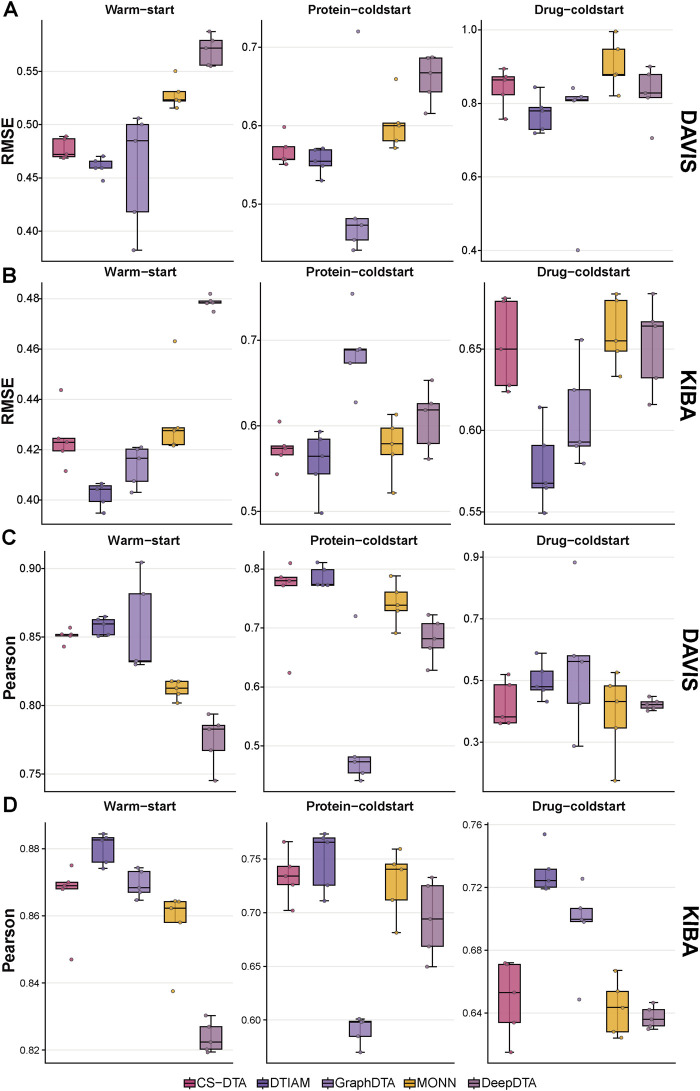
Performance comparison of CS-DTA and baseline models under different data-splitting settings. Boxplots show the predictive performance of CS-DTA, DTIAM, GraphDTA, MONN, and DeepDTA on the Davis and KIBA datasets under warm-start, protein-coldstart, and drug-coldstart settings. **(A)** Root Mean Square Error (RMSE) on Davis. **(B)** RMSE on KIBA. **(C)** Pearson correlation on Davis. **(D)** Pearson correlation on KIBA. Center lines indicate medians, boxes indicate interquartile ranges, whiskers represent non-outlier ranges, and dots denote individual split results from five repeated runs.

Under the standard in-distribution warm-start evaluation, architectures integrating LLMs—specifically CS-DTA and DTIAM—exhibited dominant predictive performance (Figure 2; Supplementary Table S3). CS-DTA achieved high Pearson correlations (Davis: 0.8510 ± 0.0051; KIBA: 0.8658 ± 0.0108) and consistently low Root Mean Square Error (RMSE) values (Davis: 0.4773 ± 0.0097; KIBA: 0.4244 ± 0.0119) (Figure 2A). Similarly, the LLM-derived DTIAM demonstrated top-tier performance (Pearson of 0.8580 ± 0.0064 and RMSE of 0.4604 ± 0.0087 on Davis). Both LLM-driven frameworks significantly outperformed classical architectures such as DeepDTA and the multi-objective MONN. This substantial performance gap suggests that the rich prior knowledge embedded in massive pre-training data provides a much more robust and generalizable representational foundation.

When evaluated under the protein-coldstart setting, traditional architectures revealed distinct vulnerabilities. DeepDTA and MONN experienced notable performance declines, while GraphDTA suffered a substantial drop in its ranking ability (with its Pearson correlation plummeting to 0.6613 ± 0.0434 on Davis and 0.5905 ± 0.0131 on KIBA). In stark contrast, the LLM-driven models maintained robust ranking capabilities and tightly bounded errors on previously unseen targets. Specifically, CS-DTA sustained highly stable metrics across both datasets, achieving Pearson correlations of 0.7544 ± 0.0743 (Davis) and 0.7342 ± 0.0234 (KIBA), paired with low RMSE values of 0.5672 ± 0.0192 (Davis) and 0.5729 ± 0.0221 (KIBA).

The drug-coldstart setting poses the most severe distribution shift, mimicking the screening of entirely novel chemical scaffolds. In this highly challenging scenario, conventional baselines like DeepDTA and MONN showed pronounced vulnerability, with MONN’s Pearson dropping to 0.3926 ± 0.1390 and its RMSE surging to 0.9038 ± 0.0682 on Davis. Conversely, CS-DTA demonstrated exceptional robustness, delivering highly competitive Pearson correlations (Davis: 0.4224 ± 0.0750; KIBA: 0.6490 ± 0.0245) and corresponding RMSE distributions (Davis: 0.8424 ± 0.0539; KIBA: 0.6524 ± 0.0274) that are highly comparable to the DTIAM framework. Importantly, this competitive predictive performance is achieved with a substantially lighter model footprint. Our CS-DTA contains 89.87 million parameters in total, whereas DTIAM employs a 33-layer ESM-2 protein encoder with 650 million parameters as part of its representation pipeline. Moreover, the two frameworks also differ markedly in practical deployment form: CS-DTA is stored as a single fine-tuned checkpoint (321.08 MB), while DTIAM relies on an AutoGluon-based downstream ensemble that integrates multiple models through stacking and bagging, resulting in a substantially larger model artifact. These results suggest that CS-DTA provides a practical balance between generalization performance and computational efficiency for drug discovery applications.

To systematically assess the impact of data similarity on predictive performance, we evaluated CS-DTA under split settings with different similarity constraints. Model performance improved consistently as residual similarity between the training and test sets increased. Under the homology-controlled protein-coldstart setting, increasing the sequence identity threshold from 30% to 70% reduced the RMSE from 0.9061 ± 0.0026 to 0.7647 ± 0.0174, while the Pearson correlation coefficient increased from 0.3972 ± 0.0357 to 0.7484 ± 0.0225 (Supplementary Figure S2A, B). A similar trend was observed under the similarity-controlled drug-coldstart setting: as the Tanimoto similarity threshold increased from 30% to 50%, the RMSE decreased from 0.9942 ± 0.1132 to 0.7359 ± 0.0085, and the Pearson correlation coefficient increased from 0.2285 ± 0.0348 to 0.5217 ± 0.0139 (Supplementary Figure S2C, D). Although performance declined under more stringent similarity constraints, CS-DTA remained predictive across both remote protein and chemical space, supporting its capacity to generalize beyond closely related training examples.

### 3.3 Ablation studies

To systematically dissect the contributions of representation learning and interaction modeling, we constructed several structural variants of CS-DTA for ablation analysis (Table 1). The full model was compared with five modified variants: “Protein CNN encoder”, in which the ESM2-based protein encoder was replaced with a conventional CNN; “Drug GNN encoder”, in which ChemBERTa was replaced with a baseline GNN; “W/o attention”, in which the bidirectional cross-attention module was removed and the unimodal representations were directly concatenated for regression; and two unidirectional interaction variants, “ $L_{p}\to L_{d}$ ” and “ $L_{d}\to L_{p}$ ”, which retained only one direction of cross-modal attention. Collectively, these variants were designed to separately evaluate the roles of LLM-based, explicit interaction modeling, and interaction directionality under different generalization settings.

**TABLE 1:** Ablation study of the CS-DTA framework under varying evaluation settings.

Method	Setting	RMSE	Pearson	Spearman	CI
W/o attention	Warm-start	0.5191 (0.0130)	0.8192 (0.0074)	0.6747 (0.0047)	0.8765 (0.0044)
	Protein-coldstart	0.5820 (0.0129)	0.7656 (0.0144)	0.6301 (0.0086)	0.8480 (0.0073)
	Drug-coldstart	0.8076 (0.0374)	0.4482 (0.0682)	0.3808 (0.0849)	0.7060 (0.0435)
Protein CNN encoder	Warm-start	0.5297 (0.0204)	0.8241 (0.0119)	0.5571 (0.0140)	0.8793 (0.0072)
	Protein-coldstart	0.6131 (0.0164)	0.7364 (0.0178)	0.4899 (0.0083)	0.8348 (0.0076)
	Drug-coldstart	0.8418 (0.0775)	0.4071 (0.097)	0.2655 (0.1249)	0.6747 (0.0435)
Drug GNN encoder	Warm-start	0.5765 (0.0125)	0.7972 (0.0208)	0.4805 (0.0272)	0.8577 (0.0080)
	Protein-coldstart	0.6455 (0.0117)	0.7521 (0.0274)	0.4586 (0.0157)	0.8361 (0.0123)
	Drug-coldstart	0.9030 (0.0367)	0.3890 (0.0474)	0.2548 (0.0608)	0.6848 (0.0275)
$L_{p}\to L_{d}$	Warm-start	0.4901 (0.0074)	0.8401 (0.0057)	0.6736 (0.0071)	0.8776 (0.0049)
	Protein-coldstart	0.5606 (0.0143)	0.7846 (0.0201)	0.6276 (0.0170)	0.8477 (0.0138)
	Drug-coldstart	0.8473 (0.0652)	0.4221 (0.0913)	0.3419 (0.0481)	0.6856 (0.0352)
$L_{d}\to L_{p}$	Warm-start	0.5031 (0.0135)	0.8299 (0.0101)	0.6679 (0.0098)	0.8736 (0.0079)
	Protein-coldstart	0.6108 (0.0197)	0.7329 (0.0297)	0.5957 (0.0171)	0.8274 (0.0144)
	Drug-coldstart	0.8266 (0.0665)	0.4486 (0.1056)	0.3706 (0.0779)	0.7017 (0.0482)
Full model	Warm-start	0.4733 (0.0087)	0.8509 (0.0046)	0.6863 (0.0074)	0.8839 (0.0046)
	Protein-coldstart	0.5672 (0.0172)	0.7809 (0.0176)	0.6252 (0.0040)	0.8460 (0.0070)
	Drug-coldstart	0.8424 (0.0483)	0.4227 (0.0671)	0.3393 (0.0520)	0.6825 (0.0244)

Predictive performance was evaluated on the Davis dataset across warm-start, protein-coldstart, and drug-coldstart settings. The table compares the full bidirectional cross-attention architecture (Full model) against five structural variants. “W/o attention” denotes the baseline variant completely “without” the cross-attention module, relying exclusively on independent representation pooling. “Protein CNN encoder” and “Drug GNN encoder” represent variants where the language model-based encoders are replaced by a Convolutional Neural Network (CNN) and a Graph Neural Network (GNN), respectively. $L_{p}\to L_{d}$ and $L_{d}\to L_{p}$ indicate unidirectional cross-attention pathways from protein-to-drug and drug-to-protein. All evaluation metrics, including RMSE, Pearson correlation coefficient, Spearman correlation coefficient, and CI, are reported with the corresponding standard deviations in parentheses.

We first examined the contribution of the unimodal encoders by replacing the pretrained protein and drug modules with simpler alternatives. Both modifications led to consistent performance degradation across evaluation settings, indicating that the predictive strength of CS-DTA depends critically on robust pretrained representations. Replacing ESM2 with the Protein CNN encoder caused a clear decline, with the most evident deterioration observed under protein-coldstart evaluation, where RMSE increased from 0.5672 to 0.6131 and Spearman correlation dropped from 0.6252 to 0.4899. This result suggests that deep contextual protein representations are particularly important when the model must generalize to previously unseen targets. The effect of replacing ChemBERTa with the Drug GNN encoder was even more pronounced. Performance decreased across all settings, and the largest degradation appeared under drug-coldstart, where RMSE rose to 0.9030 and Spearman fell to 0.2548. These results indicate that strong LLM-based encoders form the foundation of CS-DTA, with drug-side representation quality exerting the greatest influence when extrapolation to novel chemical space is required.

We next assessed the overall contribution of explicit interaction modeling by comparing the full model with the W/o attention variant. Under warm-start evaluation, removing the bidirectional cross-attention module led to a clear reduction in performance, with RMSE increasing from 0.4733 to 0.5191 and Pearson correlation decreasing from 0.8509 to 0.8192. This result indicates that explicit cross-modal interaction is beneficial when drugs and proteins in the test set remain within the training distribution, as it helps capture finer compound-protein dependencies beyond unimodal encoding alone. Under the two cold-start settings, however, the advantage of the interaction module became less consistent. In particular, the W/o attention variant showed comparable, and in drug-coldstart slightly better, performance than the full model on Davis. Because this pattern was not further examined under additional split protocols, we interpret it cautiously as an empirical observation rather than a definitive conclusion. Overall, these results suggest that the benefit of explicit interaction modeling is most evident under in-distribution evaluation, whereas its contribution under stricter extrapolative settings may depend more strongly on the specific split configuration.

To further examine how interaction design influences generalization, we compared the two unidirectional attention variants, $L_{p}\to L_{d}$ and $L_{d}\to L_{p}$ . Their results show that interaction directionality also affects model behavior under distribution shift. In protein-coldstart, $L_{p}\to L_{d}$ achieved the best performance among the attention-based variants, reducing RMSE to 0.5606 and yielding stronger stability than both $L_{d}\to L_{p}$ and the full bidirectional model. In contrast, under drug-coldstart, $L_{d}\to L_{p}$ performed slightly better than the full model, with RMSE improving to 0.8266 and Spearman increasing to 0.3706. These results indicate that the value of interaction modeling depends not only on whether cross-modal attention is used, but also on how cross-modal information is introduced. Structurally, the optimal interaction strategy appears to vary with the modality undergoing the dominant distribution shift. Taken together, the ablation results suggest that CS-DTA relies on two complementary factors: strong LLM representations as the primary basis for generalization, and task-dependent interaction design as a secondary mechanism for refining affinity prediction under different evaluation regimes.

### 3.4 Interpretability analysis validates the cross-modal interaction mechanism

To explore the physical plausibility underlying the model’s predictions, we conducted a case study on the non-receptor tyrosine kinase PTK6 (BRK) paired with a representative compound—Lestaurtinib (ID 126565) (Supplementary Figure S1).

On the drug side, Lestaurtinib is a K-252a-derived indolocarbazole analogue carrying a hydroxymethyl substitution, and the broader staurosporine or indolocarbazole literature shows that this scaffold typically engages protein kinases through a conserved ATP-site binding logic: the lactam-containing aromatic core reproduces canonical hinge interactions, whereas the oxygenated sugar or bridge-like substructure contributes additional contacts in the ribose-binding region (Acharya et al., 2022; Xu et al., 2004). Against this structural background, independent sliding-window occlusion identified a sharply concentrated ligand-side hotspot, and mapping this interval back to the SMILES localized the dominant signal to the oxygen-containing bridged fragment around the O-linked ring junction adjacent to the hydroxylated side chain (Supplementary Table S4). The same region was recovered by segment-level SHapley Additive exPlanations (SHAP) on the drug axis, indicating convergence between perturbation-based and attribution-based analyses. Taken together, these results support a chemically coherent interpretation in which the model is not relying on diffuse ligand-wide statistics, but instead prioritizes a compact, binding-relevant substructure embedded within the indolocarbazole scaffold. Importantly, we interpret this hotspot as evidence for the importance of the oxygenated bridge or sugar-like region in the context of the whole scaffold, rather than as proof that a single heteroatom alone determines activity.

**FIGURE 3:**
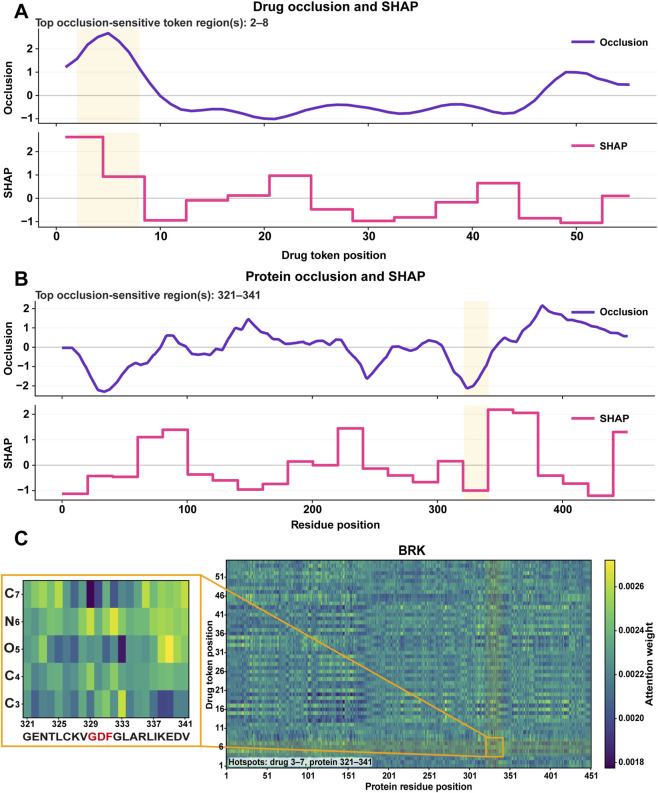
Multi-view interpretability analysis of the PTK6 (BRK)-Lestaurtinib prediction. Interpretability profiles are shown along the drug and protein axes together with the cross-attention map. **(A)** Drug-axis occlusion and SHAP profiles, with the top occlusion-sensitive region at token positions 2–8 highlighted. **(B)** Protein-axis occlusion and SHAP profiles, with the top occlusion-sensitive region at residues 321–341 highlighted. **(C)** Cross-attention heatmap between drug tokens and BRK residues, with a zoomed-in view of the local attention hotspot linking drug positions 3–7 and protein residues 321–341. The inset shows the corresponding ligand atoms and protein residues, with the DFG motif highlighted in red.

On the protein side, residue-wise occlusion revealed a highly non-uniform sensitivity landscape, with the top interval concentrated at BRK residues 321–341 (Figure 3B). This segment lies fully within the annotated PTK6 kinase domain (191–445) and directly spans the DFG motif at Asp330–Phe331–Gly332, while lying immediately adjacent to Tyr342 in the activation loop, a regulatory autophosphorylation site with documented structural relevance in PTK6 (Qiu et al., 2018; Thakur et al., 2017). Available PTK6 crystal structures further show that inhibitor recognition is organized around the ATP-binding cleft, hinge region, DFG motif, and activation-loop conformation, supporting the view that the model is concentrating on catalytically meaningful sequence determinants rather than arbitrary local patterns (Supplementary Table S5). Therefore, the enrichment of attribution signal within residues 321–341 provides biologically plausible evidence that the predictor has learned kinase-domain features relevant to ATP-competitive inhibitor recognition.

To examine how CS-DTA couples these unimodal cues, we extracted drug-to-protein ( d→p ) cross-attention weights and constructed a token-by-residue interaction map (Figure 3C). The attention heatmap exhibits structured, localized interaction blocks rather than a uniform distribution. Notably, the ligand token hotspot co-localizes with rows of elevated attention intensity, while the kinase-domain hotspot corresponds to residue columns receiving higher cumulative attention, indicating that the interaction module preferentially links the salient ligand fragment to a confined region within the catalytic domain. Taken together, these tripartite analyses support a coherent mechanism: CS-DTA leverages cross-attention to associate a compact ligand fragment with a limited set of kinase-domain residues to drive affinity prediction. We emphasize that these regions reflect model-reliant evidence in the learned decision process and should be interpreted as prioritized candidates for downstream validation, rather than definitive assertions of a precise physical binding pose.

### 3.5 Downstream screening demonstrates target-specific kinase prioritization

**FIGURE 4:**
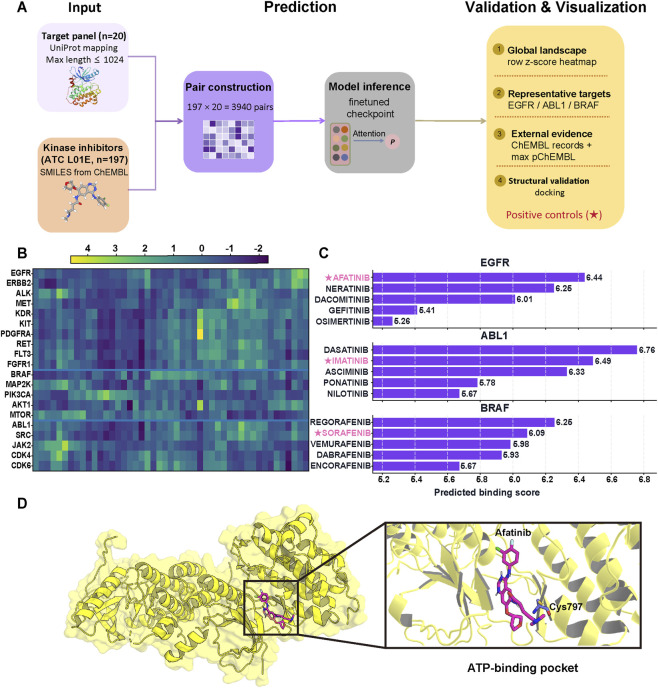
Downstream application of the CS-DTA framework in an oncology kinase screening setting. The figure summarizes the downstream screening workflow and representative analysis outputs. **(A)** Schematic representation of the CS-DTA screening pipeline. The framework systematically evaluated 3,940 potential interactions across 20 kinases and a curated library of 197 inhibitors. The workflow encompasses data integration, model-based affinity prediction, and subsequent verification. **(B)** Row z-score heatmap of predicted binding scores across the kinase panel, where color gradients reflect the relative strength of predicted interactions. **(C)** Representative target-wise rankings for EGFR, ABL1, and BRAF, with starred compounds indicating positive controls. **(D)** Structural visualization of the docked EGFR-afatinib complex in the ATP-binding pocket, with Cys797 annotated.

This oncology-oriented downstream evaluation is motivated by the clinical need to prioritize therapeutically relevant targets across diverse cancer contexts (Zhuo et al., 2022a; Zhuo et al., 2022b). We curated a panel of 20 high-value therapeutic kinases (Supplementary Table S6) and a library of 197 FDA-approved small-molecule kinase inhibitors (Supplementary Table S7), generating 3,940 candidate pairs for inference (Figure 4A). The drug-target interaction landscape, visualized via a row-wise z-score heatmap (Figure 4B), exhibits distinct, target-specific preference patterns rather than non-specific score inflation. Notably, the model successfully reconstructed biologically coherent structural relationships. Closely related receptor tyrosine kinases (RTKs), such as EGFR and ERBB2, displayed highly similar preference patterns, reflecting their shared catalytic pocket architectures and overlapping chemotypes. Conversely, non-receptor tyrosine kinases (e.g., ABL1, SRC, JAK2) exhibited divergent preference signatures. This confirms that the LLM-based models effectively capture target-specific structural determinants beyond broad-spectrum affinities.

#### 3.5.1 Target-wise rankings reliably recover established pharmacological relationships

Target-wise ranking analyses further demonstrated the framework’s capacity to recover established clinical relationships with high fidelity. For canonical oncology targets (EGFR, ABL1, and BRAF), the highest-ranked compounds were consistently enriched for well-known, mechanism-specific inhibitors (Figure 4C). For EGFR, the model prioritized clinically validated ERBB-family inhibitors, including afatinib, neratinib, and osimertinib. For ABL1, the foundational therapy imatinib and subsequent-generation inhibitors (e.g., dasatinib, ponatinib) secured top rankings, demonstrating successful recognition of diverse binding mechanisms ranging from ATP-competitive to allosteric inhibition. For BRAF, highly selective inhibitors like vemurafenib and dabrafenib were accurately isolated.

Crucially, these computational predictions are unequivocally corroborated by empirical bioactivity data from the ChEMBL database. The highly ranked EGFR-afatinib, ABL1-imatinib, and BRAF-sorafenib pairs exhibit extensive experimental footprints with peak pChEMBL values of 10.1, 9.0, and 9.0, respectively (Supplementary Table S8). This robust experimental consensus validates the accuracy of our predicted affinity scores.

#### 3.5.2 Structural validation confirms binding plausibility within the catalytic pocket

To evaluate the structural plausibility of the model’s top-ranked prediction, we performed structure-based validation on the representative EGFR-afatinib complex (Figure 4D). Using a high-resolution co-crystal structure of the EGFR kinase domain (PDB ID: 4G5P) as the receptor template, molecular docking yielded a favorable docking score of −8.5 kcal/mol. The predicted binding pose was located within the catalytic ATP-binding pocket and adopted a reasonable orientation near Cys797, the known covalent target residue of afatinib. Because afatinib acts as an irreversible covalent inhibitor, the present docking analysis was used to evaluate a plausible non-covalent pre-reaction binding pose rather than to reconstruct the final covalent adduct. Overall, these results provide supportive structural validation for the physical plausibility of the predicted EGFR-afatinib interaction.

#### 3.5.3 External validation on a non-kinase target panel

To further examine whether the learned representation of CS-DTA retains transferability beyond kinase-centric benchmarks, we selected four oncology-relevant non-kinase targets for analysis: PARP1 (UniProt accession: P09874), ESR1 (UniProt accession: P03372), CYP19A1 (UniProt accession: P11511), and AR (UniProt accession: P10275). These targets represent distinct non-kinase classes, including a DNA-repair-associated enzyme (PARP1), a steroidogenic enzyme (CYP19A1), and nuclear hormone receptors (ESR1 and AR) (Chumsri et al., 2011; Fujita and Nonomura, 2019; Johnston and Cheung, 2018; Zhu and Shi, 2025), and are supported by curated ligand annotations. We scored all target-ligand combinations within the panel and evaluated whether annotated reference ligands could be recovered near the top of the ranked lists. The results demonstrate that the model successfully prioritized known therapeutic ligands within the top-tier rankings (ideally between positions 2 and 8), particularly for the ESR1 target, where the therapeutic agent was accurately predicted as position 2, demonstrating extremely high predictive accuracy. These findings strongly demonstrate that the molecular features learned by the model are not limited to the kinase domain, but possess the generalization potential to preferentially recognize active ligands in a broader protein space.

## 4 Discussion

In this study, we developed CS-DTA, a modular encode–interact–predict framework for drug–target affinity prediction designed to operate under strict cold-start conditions, a setting intended to approximate practically relevant cold-start conditions in drug discovery. By integrating domain-specific large language models—ChemBERTa for small molecules and ESM2 for proteins—with an adaptable cross-modal interaction module, CS-DTA establishes a transferable representation foundation for modeling compound–protein relationships. Systematic benchmarking on the Davis and KIBA datasets demonstrated that the proposed framework achieves state-of-the-art predictive robustness across warm-start, protein-coldstart, and drug-coldstart evaluation settings. CS-DTA maintained stable ranking ability and bounded prediction errors even when evaluated on previously unseen compounds or protein sequences. Ablation analyses further showed that strong pretrained unimodal representations are the primary basis of generalization in CS-DTA, whereas the contribution of explicit interaction modeling is more split-dependent and varies with the way cross-modal information is introduced. Beyond benchmark evaluation, downstream virtual screening across an oncology kinase panel successfully rediscovered well-established drug-target relationships and was supported by molecular docking analyses, highlighting the potential of CS-DTA as a practical tool for large-scale therapeutic prioritization.

Beyond significant predictive generalization across rigorous data splitting strategies, the interpretability analyses indicate that CS-DTA is not merely an unconstrained black-box predictor, but a model capable of capturing local interaction determinants that are meaningful from a structural-biology perspective. This interpretation is consistent with prior studies in compound-protein interaction (CPI) and DTA prediction, which suggest that informative models should identify interaction-relevant atom-residue patterns rather than rely solely on global pair representations. For example, MONN linked affinity prediction with pairwise non-covalent interaction modeling (Li et al., 2020), while ColdstartCPI emphasized amino acid–substructure contributions as a key basis for robust cold-start generalization (Zhao Q. et al., 2025). Recent work has further shown that interpretability becomes more convincing when statistically highlighted regions are supported by structural evidence. In SCAGE, highly weighted substructures were consistent with docking-identified sensitive sites (Qiao et al., 2025). In our study, the agreement among protein-side hotspots, drug-side contribution maps, and docking analyses provides convergent evidence that CS-DTA captures biologically and chemically plausible determinants of affinity. Although such observations should be regarded as support for structural plausibility rather than definitive mechanistic proof, they nonetheless suggest that CS-DTA can serve as both an affinity predictor and a useful hypothesis-generation tool for downstream virtual screening.

Despite these encouraging results, several limitations remain and point toward promising directions for future work. First, the current framework relies primarily on sequence and string-based representations and therefore does not explicitly model conformational dynamics, induced-fit effects, or solvent-mediated interactions that often play critical roles in protein-ligand recognition (Michino et al., 2025; Sadybekov and Katritch, 2023). Incorporating structural context, such as AlphaFold-derived protein structures or ligand conformational ensembles, may further improve predictive realism (Abramson et al., 2024; Jumper et al., 2021). Second, although strict cold-start evaluation reduces potential data leakage, broader validation using external datasets or prospective time-split benchmarks would provide stronger evidence of real-world generalization (Joeres et al., 2025). Future work could also explore uncertainty-aware prediction and active learning strategies to guide experimental prioritization (Xiang et al., 2022; Zhao Y. et al., 2025). From an application perspective, CS-DTA is particularly well suited as a front-end prioritization module in multi-stage drug discovery pipelines, where rapid sequence-based screening can be combined with downstream docking, molecular dynamics simulations, and experimental validation.

## Data Availability

The original contributions presented in the study, including the datasets and source code, can be found in the GitHub repository at the following link: https://github.com/firain-bear/CS-DTA.
