# Supplementary material for: CS-DTA: a language model-driven framework for robust drug-target affinity prediction under strict cold-start scenarios
Source: Front Chem. 2026 Apr 28;14:1834317. doi: 10.3389/fchem.2026.1834317 (PMC13161074; doi:10.3389/fchem.2026.1834317)
Supplement: Supplementary file 1 [file DataSheet1.docx]

Supplementary Material

# Supplementary Figures and Tables

## Supplementary Figures

##
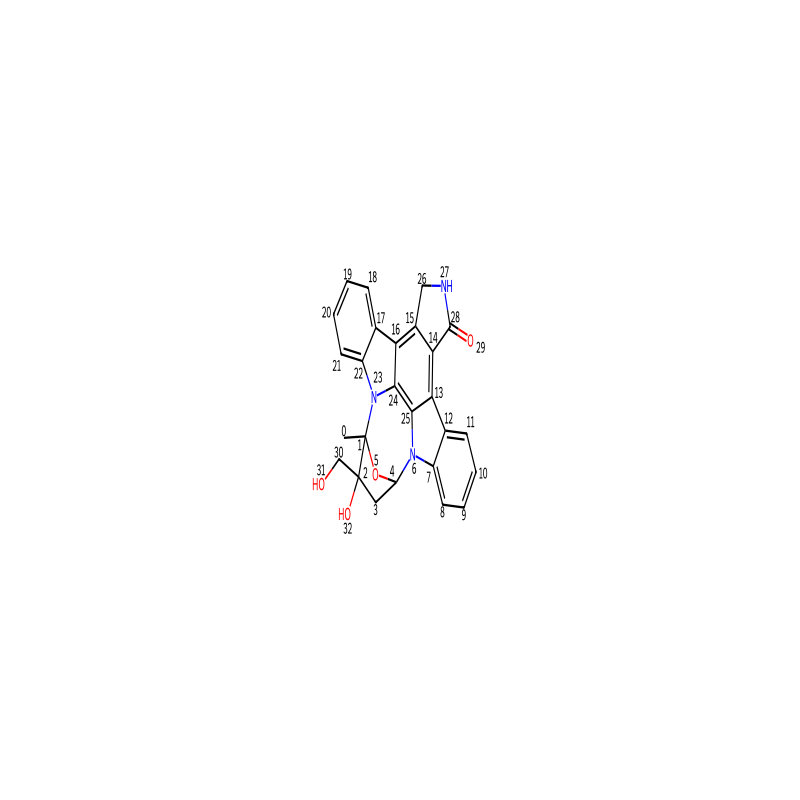


## **Supplementary Figure 1.** **Atom-indexed 2D structure of Lestaurtinib used in the BRK case study.**

The ligand is shown as a 2D chemical diagram with atom indices labeled to enable unambiguous mapping between model-derived token attributions and molecular substructures. Blue labels denote nitrogen atoms, and red labels denote oxygen atoms (including hydroxyl and carbonyl oxygens); all other atoms are carbon and hydrogen shown in black. Atom numbering follows the RDkit-generated atom order used in our downstream annotation and interpretability analyses.


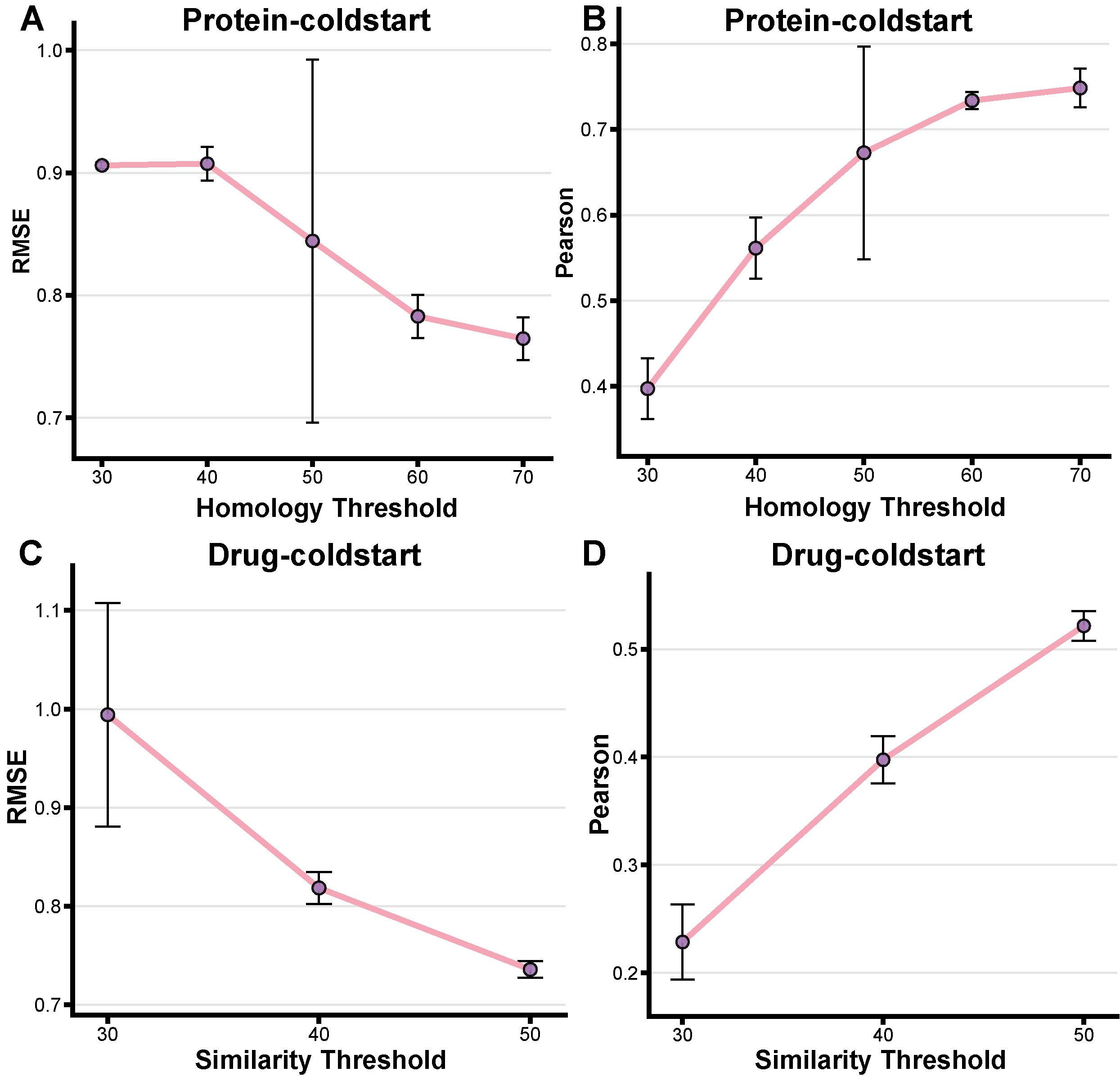


**Supplementary Figure 2. Performance of CS-DTA under stricter similarity-controlled cold-start settings.**

(A,B) Performance under protein-coldstart on the KIBA dataset across protein homology thresholds of 0.3, 0.4, 0.5, 0.6, 0.7, measured by RMSE (A) and Pearson correlation (B). (C,D) Performance under drug-coldstart on the Davis dataset across chemical similarity thresholds of 0.30, 0.40, and 0.50, measured by RMSE (C) and Pearson correlation (D). Lower thresholds correspond to stricter splits with reduced train-test similarity, whereas higher thresholds define relatively milder settings. Data points indicate mean performance across repeated runs, and error bars represent standard deviations.


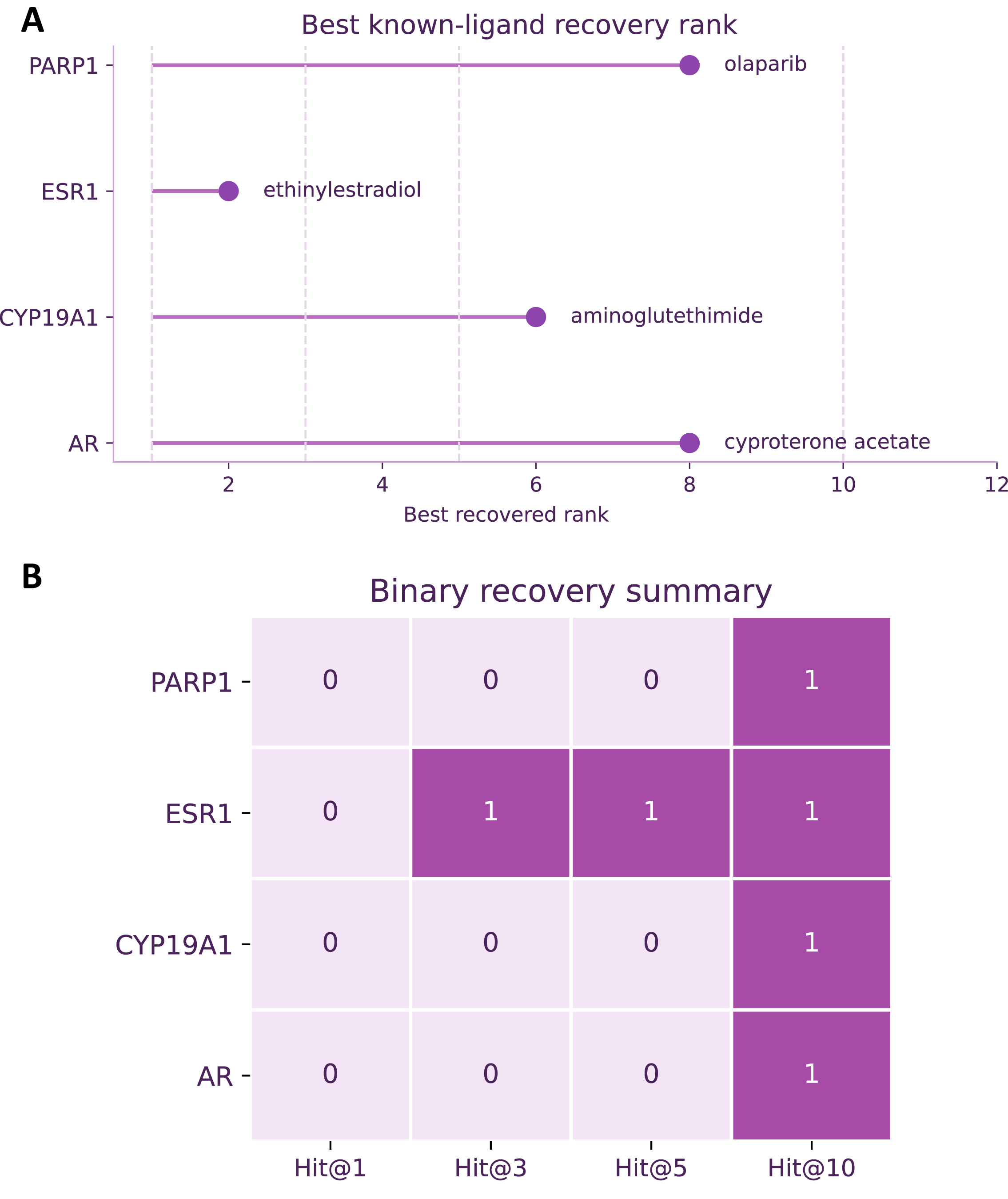


**Supplementary Figure 3.** **External validation of CS-DTA on a non-kinase target panel.**

(A) Best recovered rank of curated known ligands for four therapeutically relevant non-kinase targets, including PARP1, ESR1, CYP19A1, and AR. Each point indicates the highest-ranked recovered known ligand for the corresponding target, and the adjacent label denotes the identity of that ligand. Dashed vertical lines mark rank thresholds of 1, 3, 5, and 10.

(B) Binary recovery summary across rank thresholds. A value of 1 indicates that at least one known ligand for the corresponding target was recovered within the top K predictions, whereas 0 indicates no recovery within that range. Consistent with panel A, ESR1 showed the strongest ranking concentration, achieving recovery within the top1 (Hit@1), top 3 (Hit@3), top 5 (Hit@5), and top 10 (Hit@10) predictions, whereas PARP1, CYP19A1, and AR each showed recovery at the top-10 level only.

## Supplementary Tables

**Supplementary Table S1 Drug-side nearest-neighbor chemical similarity audit of the released drug-coldstart split**

| Dataset | Fold | Scaffold overlap rate | Mean nearest similarity | Median nearest similarity | p95 nearest similarity |
| --- | --- | --- | --- | --- | --- |
| Davis | 0 | 0 | 0.3629 | 0.3286 | 0.6243 |
|  | 1 | 0 | 0.3326 | 0.2597 | 0.5935 |
|  | 2 | 0 | 0.3757 | 0.3254 | 0.7146 |
|  | 3 | 0 | 0.3554 | 0.3269 | 0.587 |
|  | 4 | 0 | 0.3015 | 0.2523 | 0.6354 |
|  | Full | 0 | 0.3456 (0.0292) | 0.2985 (0.0390) | 0.6310 (0.0510) |
| KIBA | 0 | 0.5461 | 0.6381 | 0.6981 | 0.9385 |
|  | 1 | 0.5261 | 0.6521 | 0.7015 | 0.9689 |
|  | 2 | 0.5592 | 0.6629 | 0.7075 | 0.9198 |
|  | 3 | 0.5616 | 0.6577 | 0.7154 | 0.9147 |
|  | 4 | 0.5308 | 0.6457 | 0.6991 | 0.9385 |
|  | Full | 0.5448 (0.0161) | 0.6513 (0.0098) | 0.7043 (0.0072) | 0.9361 (0.0213) |

**Supplementary Table S2 BLASTp-based nearest-neighbor sequence similarity audit of the released protein-coldstart split.**

| Dataset | Fold | Train unique proteins | Test unique proteins | Mean best hit pident | Prop pident gt 50 |
| --- | --- | --- | --- | --- | --- |
| Davis | 0 | 353 | 89 | 0.6409 | 0.6629 |
|  | 1 | 353 | 89 | 0.6473 | 0.7191 |
|  | 2 | 354 | 89 | 0.6423 | 0.6477 |
|  | 3 | 354 | 89 | 0.6122 | 0.6552 |
|  | 4 | 354 | 89 | 0.6399 | 0.6818 |
|  | Full | - | - | 0.6365 (0.1389) | 0.6733 (0.0286) |
| KIBA | 0 | 183 | 46 | 0.5261 | 0.4565 |
|  | 1 | 183 | 46 | 0.5959 | 0.6667 |
|  | 2 | 183 | 46 | 0.5798 | 0.5333 |
|  | 3 | 183 | 46 | 0.5610 | 0.6222 |
|  | 4 | 184 | 45 | 0.5632 | 0.500 |
|  | Full | - | - | 0.5652 (0.0260) | 0.5557 (0.0869) |

**Supplementary Table S3. Source data used for baseline performance comparison across datasets and evaluation settings.**

| Dataset | Setting | Models | RMSE | Pearson |
| --- | --- | --- | --- | --- |
| Davis | Warm-start | DeepDTA | 0.5670 (0.0142) | 0.7749 (0.0191) |
|  |  | MONN | 0.5286 (0.0113) | 0.8118 (0.0067) |
|  |  | GraphDTA | 0.4583 (0.0551) | 0.8295 (0.0106) |
|  |  | DTIAM | 0.4604 (0.0087) | 0.8580 (0.0064) |
|  |  | CS-DTA | 0.4773 (0.0097) | 0.8510 (0.0051) |
|  | Protein-coldstart | DeepDTA | 0.6599 (0.0305) | 0.6813 (0.0367) |
|  |  | MONN | 0.6029 (0.0342) | 0.7417 (0.0361) |
|  |  | GraphDTA | 0.7356 (0.1876) | 0.6613 (0.0434) |
|  |  | DTIAM | 0.5546 (0.0167) | 0.7858 (0.0181) |
|  |  | CS-DTA | 0.5672 (0.0192) | 0.7544 (0.0743) |
|  | Drug-coldstart | DeepDTA | 0.8256 (0.0756) | 0.4229 (0.0180) |
|  |  | MONN | 0.9038 (0.0682) | 0.3926 (0.1390) |
|  |  | GraphDTA | 0.5140 (0.1163) | 0.4965 (0.0492) |
|  |  | DTIAM | 0.7722 (0.0502) | 0.5003 (0.0608) |
|  |  | CS-DTA | 0.8424 (0.0539) | 0.4224 (0.0750) |
| KIBA | Warm-start | DeepDTA | 0.4785 (0.0025) | 0.8238 (0.0046) |
|  |  | MONN | 0.4326 (0.0173) | 0.8696 (0.0075) |
|  |  | GraphDTA | 0.4137 (0.0080) | 0.8695 (0.0041) |
|  |  | DTIAM | 0.4021 (0.0050) | 0.8801 (0.0047) |
|  |  | CS-DTA | 0.4244 (0.0119) | 0.8658 (0.0108) |
|  | Protein-coldstart | DeepDTA | 0.6077 (0.0371) | 0.6940 (0.0356) |
|  |  | MONN | 0.5756 (0.0350) | 0.6746 (0.0378) |
|  |  | GraphDTA | 0.6869 (0.0455) | 0.5905 (0.0131) |
|  |  | DTIAM | 0.5568 (0.0380) | 0.7490 (0.0287) |
|  |  | CS-DTA | 0.5729 (0.0221) | 0.7342 (0.0234) |
|  | Drug-coldstart | DeepDTA | 0.6526 (0.0277) | 0.6372 (0.0710) |
|  |  | MONN | 0.6602 (0.0214) | 0.6662 (0.0176) |
|  |  | GraphDTA | 0.6088 (0.0312) | 0.6957 (0.0285) |
|  |  | DTIAM | 0.5773 (0.0254) | 0.7299 (0.0144) |
|  |  | CS-DTA | 0.6524 (0.0274) | 0.6490 (0.0245) |

**Supplementary Table S4. Mapping of interpretability hotspots to structural annotations on the drug side**

| Item | Hotspot definition | Hotspot interval | SMILES | SMILES substring | Structural interpretation |
| --- | --- | --- | --- | --- | --- |
| Ligand (126565) | Top occlusion-sensitive window (ranked by $\vert\Delta y\vert$) | 2–8 | [2:11] | 12C(CC(O1 | Oxygen-containing fragment around an **O-linked ring junction** adjacent to the hydroxylated side chain (see Supplementary Fig. S1 for atom-indexed 2D structure). |

**Supplementary Table S5. Mapping of interpretability hotspots to protein feature annotations on the protein side**

| Target | Hotspot definition | Hotspot interval (residue pos.) | UniProt feature type | Feature description | Feature  start-end | Overlap length | Overlap fraction |
| --- | --- | --- | --- | --- | --- | --- | --- |
| PTK6 (BRK) | Top occlusion-sensitive window (ranked by $\vert\Delta y\vert$) | 321-341 | Domain | Protein kinase | 191–445 | 21 | 1.0 |
| PTK6 (BRK) | Same hotspot | 321–341 | α helix | - | 322–324 | 3 | 0.1429 |
| PTK6 (BRK) | Same hotspot | 321–341 | β strand | - | 326–328 | 3 | 0.1429 |

**Supplementary Table S6. Oncology kinase target panel used in downstream screening**

| Gene symbol | UniProtKB accession | Protein name |
| --- | --- | --- |
| EGFR | C9JYS6 | Receptor protein-tyrosine kinase |
| ERBB2 | X5DNK3 | Receptor tyrosine-protein kinase erbB-2 |
| ALK | Q9UM73 | ALK tyrosine kinase receptor |
| MET | A0PJF7 | MET protein |
| KDR | A0A0U2VU55 | Kinase insert domain receptor |
| KIT | A0A8I5KRE7 | Mast/stem cell growth factor receptor Kit |
| PDGFRA | D6RDX0 | Platelet-derived growth factor receptor-like protein |
| RET | Q9UE13 | Ret proto-oncogene |
| FLT3 | A0A6G6D045 | FLT3 |
| FGFR1 | A0A3B3ISD1 | Fibroblast growth factor receptor |
| BRAF | A0A1X9T4I6 | B-Raf serine/threonine-protein |
| MAP2K1 | H3BRW9 | Dual specificity mitogen-activated protein kinase kinase 1 |
| PIK3CA | A0A2P0XI22 | Phosphatidylinositol 4,5-bisphosphate 3-kinase catalytic subunit alpha isoform |
| AKT1 | A0AAQ5BHJ3 | non-specific serine/threonine protein kinase |
| MTOR | A0A8V8TQN3 | Serine/threonine-protein kinase mTOR |
| CDK4 | F8VTV8 | Cyclin-dependent kinase 4 |
| CDK6 | Q00534 | Cyclin-dependent kinase 6 |
| ABL1 | Q13689 | ABL1 protein |
| SRC | Q71UK5 | Tyrosine kinase pp60c-src |
| JAK2 | Q506Q0 | Tyrosine-protein kinase |

**Supplementary Table S7. Drug library used in downstream screening**

| Drug accession | Drug name | Smiles length |
| --- | --- | --- |
| CHEMBL413 | SIROLIMUS | 190 |
| CHEMBL417 | EPIRUBICIN | 98 |
| CHEMBL427 | MECHLORETHAMINE | 12 |
| CHEMBL38 | TRETINOIN | 47 |
| CHEMBL43 | AMSACRINE | 44 |
| CHEMBL58 | MITOXANTRONE | 50 |
| CHEMBL467 | HYDROXYUREA | 8 |
| CHEMBL476 | DACARBAZINE | 26 |
| CHEMBL481 | IRINOTECAN | 80 |
| CHEMBL84 | TOPOTECAN | 60 |
| CHEMBL428647 | PACLITAXEL | 160 |
| CHEMBL513 | CARMUSTINE | 20 |
| CHEMBL514 | LOMUSTINE | 24 |
| CHEMBL515 | CHLORAMBUCIL | 30 |
| CHEMBL98 | VORINOSTAT | 27 |
| CHEMBL535 | SUNITINIB | 55 |
| CHEMBL105 | MITOMYCIN | 67 |
| CHEMBL553 | ERLOTINIB | 42 |
| CHEMBL554 | LAPATINIB | 68 |
| CHEMBL118 | CELECOXIB | 50 |
| CHEMBL601 | AMINOLEVULINIC ACID | 15 |
| CHEMBL671 | THIOTEPA | 22 |
| CHEMBL705 | ALITRETINOIN | 47 |
| CHEMBL24828 | VANDETANIB | 51 |
| CHEMBL727 | THIOGUANINE | 24 |
| CHEMBL760 | ANAGRELIDE | 30 |
| CHEMBL34259 | METHOTREXATE | 63 |
| CHEMBL803 | CYTARABINE | 49 |
| CHEMBL810 | TEMOZOLOMIDE | 24 |
| CHEMBL820 | BUSULFAN | 26 |
| CHEMBL830 | EFLORNITHINE | 21 |
| CHEMBL288441 | BOSUTINIB | 59 |
| CHEMBL852 | MELPHALAN | 37 |
| CHEMBL44657 | ETOPOSIDE | 116 |
| CHEMBL46286 | OMACETAXINE MEPESUCCINATE | 83 |
| CHEMBL159 | VINBLASTINE | 147 |
| CHEMBL888 | GEMCITABINE | 47 |
| CHEMBL53463 | DOXORUBICIN | 97 |
| CHEMBL917 | FLOXURIDINE | 47 |
| CHEMBL939 | GEFITINIB | 46 |
| CHEMBL941 | IMATINIB | 59 |
| CHEMBL1023 | BEXAROTENE | 48 |
| CHEMBL1024 | IFOSFAMIDE | 21 |
| CHEMBL90555 | VINCRISTINE | 148 |
| CHEMBL313972 | MASOPROCOL | 47 |
| CHEMBL1117 | IDARUBICIN | 93 |
| CHEMBL1129 | TRIFLURIDINE | 54 |
| CHEMBL1321 | PROCARBAZINE | 25 |
| CHEMBL1336 | SORAFENIB | 57 |
| CHEMBL167731 | PIXANTRONE | 36 |
| CHEMBL180022 | NERATINIB | 66 |
| CHEMBL189963 | PALBOCICLIB | 55 |
| CHEMBL178 | DAUNORUBICIN | 96 |
| CHEMBL1421 | DASATINIB ANHYDROUS | 55 |
| CHEMBL185 | FLUOROURACIL | 23 |
| CHEMBL225071 | RALTITREXED | 66 |
| CHEMBL225072 | PEMETREXED | 66 |
| CHEMBL1455 | ALTRETAMINE | 27 |
| CHEMBL1488 | URACIL MUSTARD | 25 |
| CHEMBL1489 | AZACITIDINE | 48 |
| CHEMBL255863 | NILOTINIB | 70 |
| CHEMBL408513 | BELINOSTAT | 40 |
| CHEMBL455186 | TREOSULFAN | 41 |
| CHEMBL452231 | TENIPOSIDE | 122 |
| CHEMBL1580 | PENTOSTATIN | 46 |
| CHEMBL1585 | PIPOBROMAN | 28 |
| CHEMBL487253 | BENDAMUSTINE | 38 |
| CHEMBL473417 | VISMODEGIB | 53 |
| CHEMBL1619 | CLADRIBINE | 45 |
| CHEMBL500576 | TEMOPORFIN | 101 |
| CHEMBL521686 | OLAPARIB | 58 |
| CHEMBL451930 | PLITIDEPSIN | 203 |
| CHEMBL477772 | PAZOPANIB | 55 |
| CHEMBL450449 | TRABECTEDIN | 125 |
| CHEMBL483254 | PANOBINOSTAT | 43 |
| CHEMBL1670 | MITOTANE | 32 |
| CHEMBL502835 | NINTEDANIB | 72 |
| CHEMBL553025 | VINORELBINE | 141 |
| CHEMBL576982 | QUIZARTINIB | 68 |
| CHEMBL1750 | CLOFARABINE | 52 |
| CHEMBL1773 | CAPECITABINE | 59 |
| CHEMBL601719 | CRIZOTINIB | 56 |
| CHEMBL608533 | MIDOSTAURIN | 91 |
| CHEMBL1078178 | MOMELOTINIB | 50 |
| CHEMBL1096562 | METHYL AMINOLEVULINATE | 16 |
| CHEMBL1096882 | FLUDARABINE PHOSPHATE | 60 |
| CHEMBL1096885 | VALRUBICIN | 120 |
| CHEMBL1094636 | NIRAPARIB | 45 |
| CHEMBL1171837 | PONATINIB | 65 |
| CHEMBL1173655 | AFATINIB | 61 |
| CHEMBL1173055 | RUCAPARIB | 41 |
| CHEMBL1425 | MERCAPTOPURINE | 23 |
| CHEMBL88 | CYCLOPHOSPHAMIDE | 25 |
| CHEMBL1201112 | NELARABINE | 52 |
| CHEMBL1201129 | DECITABINE | 42 |
| CHEMBL1201182 | TEMSIROLIMUS | 205 |
| CHEMBL1201746 | PRALATREXATE | 66 |
| CHEMBL1201748 | CABAZITAXEL | 156 |
| CHEMBL1201752 | IXABEPILONE | 99 |
| CHEMBL1229517 | VEMURAFENIB | 63 |
| CHEMBL1237054 | PLICAMYCIN | 259 |
| CHEMBL1287853 | FEDRATINIB | 61 |
| CHEMBL1289494 | TIVOZANIB | 52 |
| CHEMBL1289601 | LENVATINIB | 51 |
| CHEMBL1289926 | AXITINIB | 47 |
| CHEMBL1614701 | SELUMETINIB | 46 |
| CHEMBL1683590 | ERIBULIN | 174 |
| CHEMBL1738797 | ALECTINIB | 63 |
| CHEMBL1770916 | NIROGACESTAT | 68 |
| CHEMBL1789941 | RUXOLITINIB | 44 |
| CHEMBL1852688 | INFIGRATINIB | 65 |
| CHEMBL1873475 | IBRUTINIB | 61 |
| CHEMBL1908360 | EVEROLIMUS | 193 |
| CHEMBL1946170 | REGORAFENIB | 60 |
| CHEMBL1977579 | STREPTOZOCIN | 51 |
| CHEMBL1983268 | ENTRECTINIB | 71 |
| CHEMBL2028663 | DABRAFENIB | 65 |
| CHEMBL2035187 | PACRITINIB | 61 |
| CHEMBL2043437 | GLASDEGIB | 57 |
| CHEMBL2103875 | TRAMETINIB | 72 |
| CHEMBL2105717 | CABOZANTINIB | 61 |
| CHEMBL2110732 | DACOMITINIB | 56 |
| CHEMBL2105737 | SONIDEGIB | 71 |
| CHEMBL2110725 | VINFLUNINE | 151 |
| CHEMBL2146883 | COBIMETINIB | 56 |
| CHEMBL2216870 | IDELALISIB | 56 |
| CHEMBL2325741 | CAPIVASERTIB | 59 |
| CHEMBL2396661 | ALPELISIB | 59 |
| CHEMBL2403108 | CERITINIB | 65 |
| CHEMBL3039502 | DUVELISIB | 56 |
| CHEMBL3137309 | VENETOCLAX | 118 |
| CHEMBL3137320 | TALAZOPARIB | 59 |
| CHEMBL3187723 | BINIMETINIB | 45 |
| CHEMBL3188267 | CAPMATINIB | 49 |
| CHEMBL3218576 | COPANLISIB | 56 |
| CHEMBL3286830 | LORLATINIB | 57 |
| CHEMBL3301610 | ABEMACICLIB | 64 |
| CHEMBL3301612 | ENCORAFENIB | 71 |
| CHEMBL3301622 | GILTERITINIB | 65 |
| CHEMBL3353410 | OSIMERTINIB | 62 |
| CHEMBL3402762 | TEPOTINIB | 61 |
| CHEMBL3414621 | TAZEMETOSTAT | 72 |
| CHEMBL3545185 | SELINEXOR | 58 |
| CHEMBL92 | DOCETAXEL | 166 |
| CHEMBL3545311 | BRIGATINIB | 66 |
| CHEMBL3545376 | ERDAFITINIB | 54 |
| CHEMBL3701238 | FUTIBATINIB | 58 |
| CHEMBL3545110 | RIBOCICLIB SUCCINATE | 66 |
| CHEMBL3707348 | ACALABRUTINIB | 63 |
| CHEMBL3786343 | OLMUTINIB | 57 |
| CHEMBL3813873 | PEXIDARTINIB | 51 |
| CHEMBL3889654 | LAROTRECTINIB | 59 |
| CHEMBL3936761 | ZANUBRUTINIB | 66 |
| CHEMBL3948730 | UMBRALISIB | 78 |
| CHEMBL3989868 | TUCATINIB | 57 |
| CHEMBL3989908 | ENASIDENIB | 60 |
| CHEMBL3989958 | IVOSIDENIB | 82 |
| CHEMBL4071161 | TIRABRUTINIB | 65 |
| CHEMBL4204794 | AVAPRITINIB | 68 |
| CHEMBL4208229 | ASCIMINIB | 64 |
| CHEMBL4216467 | RIPRETINIB | 57 |
| CHEMBL4279047 | VORASIDENIB | 60 |
| CHEMBL4297516 | LURBINECTEDIN | 132 |
| CHEMBL4297522 | PEMIGATINIB | 61 |
| CHEMBL4297610 | OLUTASIDENIB | 50 |
| CHEMBL4298138 | REPOTRECTINIB | 51 |
| CHEMBL4303060 | MELPHALAN FLUFENAMIDE | 64 |
| CHEMBL4303214 | FRUQUINTINIB | 48 |
| CHEMBL4535757 | SOTORASIB | 81 |
| CHEMBL4558324 | LAZERTINIB | 69 |
| CHEMBL4559134 | SELPERCATINIB | 67 |
| CHEMBL4582651 | PRALSETINIB | 84 |
| CHEMBL4585668 | BELZUTIFAN | 62 |
| CHEMBL4594350 | ADAGRASIB | 81 |
| CHEMBL4650319 | MOBOCERTINIB | 75 |
| CHEMBL4650485 | PIRTOBRUTINIB | 66 |
| CHEMBL5483015 | ARSENIC TRIOXIDE | 19 |
| CHEMBL409 | BICALUTAMIDE | 57 |
| CHEMBL411 | DIETHYLSTILBESTROL | 35 |
| CHEMBL488 | AMINOGLUTETHIMIDE | 29 |
| CHEMBL83 | TAMOXIFEN | 45 |
| CHEMBL691 | ETHINYL ESTRADIOL | 55 |
| CHEMBL717 | MEDROXYPROGESTERONE ACETATE | 79 |
| CHEMBL806 | FLUTAMIDE | 43 |
| CHEMBL1274 | NILUTAMIDE | 51 |
| CHEMBL1358 | FULVESTRANT | 96 |
| CHEMBL1399 | ANASTROZOLE | 40 |
| CHEMBL1444 | LETROZOLE | 36 |
| CHEMBL1655 | TOREMIFENE | 47 |
| CHEMBL1082407 | ENZALUTAMIDE | 62 |
| CHEMBL1200374 | EXEMESTANE | 66 |
| CHEMBL1200598 | DIETHYLSTILBESTROL DIPHOSPHATE | 53 |
| CHEMBL1201139 | MEGESTROL ACETATE | 76 |
| CHEMBL1800159 | RELUGOLIX | 77 |
| CHEMBL3183409 | APALUTAMIDE | 63 |
| CHEMBL4297185 | DAROLUTAMIDE | 60 |
| CHEMBL4297509 | ELACESTRANT | 55 |

**Supplementary Table S8. Curated ChEMBL bioactivity evidence for representative positive controls.**

| Gene symbol | UniProt accession | Drug name | ChEMBL parent accession | Target ChEMBL accession | Number of activity records | Max pChEMBL | Top assay types |
| --- | --- | --- | --- | --- | --- | --- | --- |
| EGFR | P00533 | Afatinib | CHEMBL1173655 | CHEMBL203 | 200 | 10.1 | IC50, Inhibition, Kd |
| ABL1 | P00519 | Imatinib | CHEMBL941 | CHEMBL1862 | 160 | 9.0 | IC50, Kd, Inhibition |
| BRAF | P15056 | Sorafenib | CHEMBL1336 | CHEMBL5145 | 89 | 9.0 | IC50, Kd, Inhibition |

**Supplementary Table S9. Non-kinase target panel**

| Protein id | Gene symbol | UniProt accession | Protein name |
| --- | --- | --- | --- |
| T001 | PARP1 | P09874 | Poly [ADP-ribose] polymerase 1 |
| T002 | ESR1 | P03372 | Estrogen receptor alpha |
| T003 | CYP19A1 | P11511 | Aromatase |
| T004 | AR | P10275 | Androgen receptor |

**Supplementary Table S10. Compound panel for the non-kinase evaluation**

| Drug id | ChEMBL accession | Drug name |
| --- | --- | --- |
| D0001 | CHEMBL135 | 17β-ESTRADIOL |
| D0002 | CHEMBL1358 | FULVESTRANT |
| D0003 | CHEMBL83 | TAMOXIFEN |
| D0004 | CHEMBL411 | DIETHYLSTILBESTROL |
| D0005 | CHEMBL1276308 | MIFEPRISTONE |
| D0006 | CHEMBL1405 | ESTRONE |
| D0007 | CHEMBL81 | RALOXIFENE |
| D0008 | CHEMBL193482 | ESTRIOL |
| D0009 | CHEMBL1445 | FLUOXYMESTERONE |
| D0010 | CHEMBL409 | BICALUTAMIDE |
| D0011 | CHEMBL1274 | NILUTAMIDE |
| D0012 | CHEMBL139835 | CYPROTERONE ACETATE |
| D0013 | CHEMBL2355051 | CLOMIPHENE |
| D0014 | CHEMBL1399 | ANASTROZOLE |
| D0015 | CHEMBL1444 | LETROZOLE |
| D0016 | CHEMBL1082407 | ENZALUTAMIDE |
| D0017 | CHEMBL806 | FLUTAMIDE |
| D0018 | CHEMBL1201048 | DROMOSTANOLONE PROPIONATE |
| D0019 | CHEMBL1200623 | ETHYLESTRENOL |
| D0020 | CHEMBL757 | NANDROLONE |
| D0021 | CHEMBL488 | AMINOGLUTETHIMIDE |
| D0022 | CHEMBL691 | ETHINYLESTRADIOL |
| D0023 | CHEMBL1200374 | EXEMESTANE |
| D0024 | CHEMBL1170 | TESTOSTERONE PROPIONATE |
| D0025 | CHEMBL1571 | TESTOLACTONE |
| D0026 | CHEMBL2105395 | OSPEMIFENE |
| D0027 | CHEMBL46740 | BAZEDOXIFENE |
| D0028 | CHEMBL521686 | OLAPARIB |
| D0029 | CHEMBL1173055 | RUCAPARIB |
| D0030 | CHEMBL1094636 | NIRAPARIB |
| D0031 | CHEMBL9298 | FADROZOLE |
| D0032 | CHEMBL3137320 | TALAZOPARIB |
| D0033 | CHEMBL3183409 | APALUTAMIDE |
| D0034 | CHEMBL2103774 | TIBOLONE |
| D0035 | CHEMBL2079587 | STANOZOLOL |
| D0036 | CHEMBL4297185 | DAROLUTAMIDE |
| D0037 | CHEMBL3590187 | CLASCOTERONE |
| D0038 | CHEMBL1413 | CICLOPIROX |
| D0039 | CHEMBL4112930 | PAMIPARIB |
| D0040 | CHEMBL5095183 | IMLUNESTRANT |
| D0041 | CHEMBL3930624 | FLUZOPARIB |
| D0042 | CHEMBL4594421 | DEUTENZALUTAMIDE |

**Supplementary Table S11. Drug identities corresponding to the heatmap legend**

| Drug Index | Heatmap label | Drug accession |
| --- | --- | --- |
| D1 | OSIMERTINIB | CHEMBL3353410 |
| D2 | BOSUTINIB | CHEMBL288441 |
| D3 | ERDAFITINIB | CHEMBL3545376 |
| D4 | ALECTINIB | CHEMBL1738797 |
| D5 | CERITINIB | CHEMBL2403108 |
| D6 | PAZOPANIB | CHEMBL477772 |
| D7 | SELPERCATINIB | CHEMBL4559134 |
| D8 | LAROTRECTINIB | CHEMBL3889654 |
| D9 | LENVATINIB | CHEMBL1289601 |
| D10 | PALBOCICLIB | CHEMBL189963 |
| D11 | RUXOLITINIB | CHEMBL1789941 |
| D12 | MOMELOTINIB | CHEMBL1078178 |
| D13 | TEPOTINIB | CHEMBL3402762 |
| D14 | ZANUBRUTINIB | CHEMBL3936761 |
| D15 | CAPMATINIB | CHEMBL3188267 |
| D16 | PEXIDARTINIB | CHEMBL3813873 |
| D17 | FRUQUINTINIB | CHEMBL4303214 |
| D18 | CRIZOTINIB | CHEMBL601719 |
| D19 | COPANLISIB | CHEMBL3218576 |
| D20 | DACOMITINIB | CHEMBL2110732 |
| D21 | PIRTOBRUTINIB | CHEMBL4650485 |
| D22 | ALPELISIB | CHEMBL2396661 |
| D23 | UMBRALISIB | CHEMBL3948730 |
| D24 | COBIMETINIB | CHEMBL2146883 |
| D25 | ASCIMINIB | CHEMBL4208229 |
| D26 | NILOTINIB | CHEMBL255863 |
| D27 | REGORAFENIB | CHEMBL1946170 |
| D28 | SORAFENIB | CHEMBL1336 |
| D29 | NINTEDANIB | CHEMBL502835 |
| D30 | PONATINIB | CHEMBL1171837 |
| D31 | TRAMETINIB | CHEMBL2103875 |
| D32 | IMATINIB | CHEMBL941 |
| D33 | REPOTRECTINIB | CHEMBL4298138 |
| D34 | TIVOZANIB | CHEMBL1289494 |
| D35 | VANDETANIB | CHEMBL24828 |
| D36 | DASATINIB ANHYDROUS | CHEMBL1421 |
| D37 | LAZERTINIB | CHEMBL4558324 |
| D38 | PEMIGATINIB | CHEMBL4297522 |
| D39 | GILTERITINIB | CHEMBL3301622 |
| D40 | TUCATINIB | CHEMBL3989868 |
| D41 | AVAPRITINIB | CHEMBL4204794 |
| D42 | DUVELISIB | CHEMBL3039502 |
| D43 | IDELALISIB | CHEMBL2216870 |
| D44 | PACRITINIB | CHEMBL2035187 |
| D45 | NERATINIB | CHEMBL180022 |
| D46 | AFATINIB | CHEMBL1173655 |
| D47 | AXITINIB | CHEMBL1289926 |
